# Multi-omics techniques revealing the mechanism of *Polygonatum sibiricum* Huangjiu in alleviating hyperlipidemia in mice

**DOI:** 10.3389/fnut.2025.1705061

**Published:** 2025-11-27

**Authors:** Jingzhang Geng, Guotai Zhang, Jianwei Dong, Honglei Tian, Zaixiang Lou

**Affiliations:** 1 Shaanxi Province Key Laboratory of Bio-Resources, QinLing-Bashan Mountains Bioresources Comprehensive Development C. I. C., Qinba State Key Laboratory of Biological Resources and Ecological Environment, Shaanxi University of Technology, Hanzhong, China; 2 School of Life Science and Technology, Xi’an Jiaotong University, Xi’an, China; 3 College of Food and Chemical Engineering, Liuzhou Institute of Technology, Liuzhou, Yufeng, China; 4 School of Food Science and Technology, Jiangnan University, Wuxi, China

**Keywords:** *Polygonatum sibiricum* Huangjiu, hyperlipidemia, microbiomics, lipidomics, metabolic

## Abstract

**Introduction:**

Hyperlipidemia poses a serious threat to human health. However, given the significant adverse effect of drug therapy, finding safe and efficient lipid-lowering agents has become a focal point of interest. *Polygonatum sibiricum* Huangjiu (PSHJ) is hypothesized to exert lipid-lowering effects. This study examined PSHJ’s ability to alleviate hyperlipidemia in mice caused by a high-fat diet, and the regulatory mechanism was studied via gut microbiome and fecal lipidomics.

**Methods:**

A mouse model of hyperlipidemia was established using a high-fat diet. The experimental groups were administered PSHJ, and serum lipid profiles—including triglycerides (TG), total cholesterol (TC), low-density lipoprotein cholesterol (LDL-C), and high-density lipoprotein cholesterol (HDL-C)—were measured. Histopathological examinations of the liver, kidney, and small intestine were performed to assess tissue damage. The gut microbiota composition was analyzed to evaluate diversity and the abundance of short-chain fatty acids (SCFAs)-producing bacteria. Fecal lipidomics was employed to investigate alterations in lipid metabolic pathways.

**Results:**

PSHJ treatment significantly increased HDL-C levels while reducing serum TG, TC, and LDL-C levels in hyperlipidemic mice. Histological analysis revealed that PSHJ alleviated damage in the liver, kidney, and small intestine. Furthermore, PSHJ enhanced gut microbial diversity and promoted the proliferation of SCFA-producing bacteria, leading to elevated SCFA levels. Lipidomic analysis indicated that PSHJ modulated metabolic pathways related to glycerophospholipids, glycerolipids, and fatty acids, thereby facilitating the breakdown of TG and diacylglycerol (DG).

**Discussion:**

The findings suggest that polysaccharides in PSHJ function as prebiotics, enriching beneficial gut microbiota and increasing SCFA production. These SCFAs, along with polysaccharides, appear to regulate key lipid metabolic pathways, enhancing the degradation of TG and DG. This study shows that PSHJ has active components that can alleviate hyperlipidemia, thereby laying a theoretical foundation for extracting bioactive substances from Huangjiu for future medical or dietary use.

## 1 Introduction

Abnormal lipid metabolism, characterized by higher levels of triglycerides (TG), total cholesterol (TC), and low-density lipoprotein cholesterol (LDL-C) and decreased levels of HDL-C, is the cause of hyperlipidemia (1). Human health is greatly impacted by hyperlipidemia, which is a risk factor for a number of illnesses, such as atherosclerosis, type 2 diabetes (T2D), liver and kidney damage, and hypertension (2). In clinical practice, statins and fibrates are the most often used treatments for hyperlipidemia. Despite their excellent therapeutic efficacy, long-term treatment is necessary, which can lead to a number of adverse effects, ranging from vomiting and nausea to potential liver impairment (3). As a result, finding safe and efficient hypolipidemic medications has become a primary research focus.

Chinese rice wine (Huangjiu), has a brewing history that dates back more than 5,000 years and is a staple of traditional fermented foods. It is a prime example of low-alcohol grain fermentation beverage, its functional components, such as oligosaccharides, polysaccharides, peptides, organic acids, free amino acids, polyphenols, and trace minerals (4), provide advantageous effects that include hepatoprotection, gut microbiota enhancement, anti-inflammation, and anti-aging (5). Traditional Huangjiu also has the effect of lowering blood lipids. When compared to a high-fat diet (HFD), rats that drank Huangjiu plus HFD had better lipid metabolism, reduced the ratio of *Firmicutes* to *Bacteroidetes*, and improved microbiota dysbiosis (6). In mice with hyperlipidemia caused by a high-fat diet, the Huangjiu peptides T1 and T2, which were separated from Huangjiu, can reduce the aberrant buildup of liver lipids as well as the elevation of serum total cholesterol, triglycerides, and low-density lipoprotein cholesterol levels (7).

*Polygonatum sibiricum* (PS) is a dried rhizomes of perennial herbaceous plants in the *Liliaceae* family, it is one of the first varieties in China to enter the food and medicine homology, which has a long history of use as a nutritional food additive. Polysaccharides, phytosterols, triterpenoids, alkaloids, lignans, flavonoids, and other functional components of PS have been found, and it has the effects of anti-osteoporotic, anti-inflammatory, boosting immunity, and promoting sleep (8). PS polysaccharides (PSPs) are considered an important component of PS, with various biological functions such as antioxidant, antimicrobial, modulating gut microbiota, hypoglycemic, and lipid-lowering (9). Zeng et al. (10) have observed that PSPs not only lower the body weight of obese rats, but also reduce serum TC, TG, and LDL-C levels, and elevate serum HDL-C, which demonstrates that PSPs enhance lipid metabolism and modulate blood lipid levels. According to Wang et al. (11), in an HFD-induced mouse model of obesity, PSP decreased TC and TG levels, weight gain, fat deposition, and microbiome dysbiosis, as well as controlled the expression of genes linked to lipid metabolism in adipocytes. Its ability to reduce lipids is connected to improving of gut microbiota community structure, upregulation of short-chain fatty acids (SCFAs), and regulation of genes related to lipid synthesis (12).

Our previous studies have used wheat Qu added PS as a fermentation agent to produce *Polygonatum sibiricum* Huangjiu (PSHJ). It has been confirmed that the beneficial components of PS enhance the microbial diversity of wheat Qu, as well as the polysaccharide content and volatile flavor of Huangjiu. Huangjiu’s functional components are closely linked to the complex microbial metabolism in the fermentation environment (13). As a result, Jiuqu’s microbial community structure has a significant impact on Huangjiu’s flavor profile and bioactive components (14). The *Stachys sieboldii Miq.* Huangjiu prepared from *Stachys sieboldii Miq.* Jiuqu contains both the bioactive components of *Stachys sieboldii Miq.* and Huangjiu, and has a good effect on relieving hyperlipidemia (15). Thus, we expect that PSHJ will demonstrate remarkable biological functioning, owing to its higher polysaccharide content. However, to date, no meaningful research endeavors have been done to examine the biological activities of PSHJ.

We hypothesize that the polysaccharides in PSHJ act as prebiotics, which alleviate hyperlipidemia via modulating the metabolic pathways of glycerophospholipids, glycerolipids, and fatty acids through enhancing gut microbiota diversity and increasing SCFAs levels. Therefore, this study aims to elucidate the lipid-lowering capacity of PSHJ while investigating the mechanisms by integrating microbiomics and lipidomics. The effect of PSHJ on hyperlipidemia was evaluated by body weight, four blood lipid levels, and liver and kidney function indicators. The mechanism PSHJ acting on hyperlipidemia was explored through the changes in gut microbiota composition, fecal SCFA content, and lipid composition, as well as their interrelationships. It is anticipated that the study’s findings will offer a theoretical foundation and point of reference for the creation of functional Huangjiu.

## 2 Materials and methods

### 2.1 Animal experiment

Five-week-old male KM mice weighing 20 ± 2 g were acquired from Xi’an Botian Biotechnology Co., Ltd. and were kept in regular circumstances, which included a 12-h light/dark cycle, 23 ± 2 °C, and free access to food and water. After a week of acclimation, the mice were split into six groups at random, with 10 mice in each group: the blank control group (Con), the positive model group (Mol), the low-dose *Polygonatum sibiricum* Huangjiu group (Low), the medium dose *Polygonatum sibiricum* Huangjiu group (Mid), the high-dose *Polygonatum sibiricum* Huangjiu group (High), and the ordinary Huangjiu group (PTHJ). The Con group received a basic diet consistently, while the other groups were fed HFD for during the 5 weeks modeling phase, and then fed a basic diet augmented with corresponding Huangjiu the during 4 weeks dosing period. A basic diet supplemented with 8.3 mL/(kg·BW), 16.6 mL/(kg·BW), and 50 mL/(kg·BW) of PSHJ was given to the Low, Mid, and High groups, respectively. The Low, Mid, and High groups’ polysaccharide concentrations were 116.53 mg/kg, 233.06 mg/kg, and 702 mg/kg, respectively, based on Huangjiu’s polysaccharide content of 14.04 g/L. During the dosing period, Con and Mol groups were given an equivalent volume of 0.9% physiological saline, Low, Mid, and High groups were given PSHJ with corresponding polysaccharide concentrations, and PTHJ group was given sterilized ordinary Huangjiu with a polysaccharide content of 11.08 mg/mL. The volume of gavage supplied was 0.1 mL/10 g body weight. Prior to each gavage, the mice were weighed to determine the actual dose based on individual body weight. The Shaanxi University of Technology Animal Ethics Committee (SNUT2023167) approved the animal experiments, which were conducted in accordance with the Committee on Care and Use of Laboratory Animals’ guidelines. The reagents and the detection process of physiological and chemical indicators in mice were shown in the Supplementary materials.

### 2.2 Determination of microbiome in mouse feces

The procedure outlined in the Omega instructions was followed while extracting DNA. The DNA library was constructed in accordance with the guidelines provided by the manufacturer (Illumina). Based on the Illumina HiSeq 2000 platform, all samples were sequenced. To obtain clean DNA readings, bowite2 (Version 2.1.0) was utilized after multi-sample hybrid splicing was completed using Megahit (Version 1.2.9). To get low-abundance allele groups, control the spliced results, extract unspliced DNA reads, and then splice again using SPAdes (Version 3.13). The DNA readings were then subjected to a series of steps, including Bin categorization, Bin purification, Bin quantification, Bin recombination, and Bin identification, using Meta WRAP (Version 1.3.2). To predict the ORF of the splicing results, choose genes that are at least 100 bp long, and convert them into nucleic acid sequences, use Prodigal (Version 2.60). Salmon (Version 1.5.0) was used to create a particular index of the non-redundant genome, and CD-HIT (version 2.60) was used to eliminate duplicates to create a non-redundant gene set for each sample’s gene prediction findings. Ultimately, the gene length information was utilized to compute the gene abundance, which was then precisely quantified in each sample using the biphasic algorithm and the construction of a bias model (16).

### 2.3 Lipidomics analysis of mouse feces

After adding 20 ± 1 mg of the fecal sample to the numbered centrifuge tube, 200 *μ* L of water was added and vortexed for 1 min. Next, 1 mL of lipid extraction solution (methyl tert-butyl ether: methanol = 3:1, V/V) containing internal standard was added and vortexed for 15 min. After centrifuging the mixture for 10 min at 12,000 rpm and 4 °C, 200 μL of the supernatant was taken out and dried nearly dry. Then 200 μL of lipid reconstitution solution (acetonitrile: isopropanol = 1:1, V/V)was added, vortexed for 3 min, centrifuged for 3 min at 12,000 r/min, and the supernatant was taken out for analysis by LC–MS/MS.

Lipidomic analysis was carried out on a ExionLC AD UPLC-QTRAP system (SCIEX, Foster City, California, USA) with a Thermo AccucoreTM C30 column (i.d.2.1 × 100 mm, 2.6 μm). A: Acetonitrile and water (60/40, V/V) (containing 0.1% formic acid and 10 mmol/L ammonium formate), and B: acetonitrile and isopropanol (10/90, V/V) (containing 0.1% formic acid and 10 mmol/L ammonium formate) made up the mobile phase. The elution gradients used for analysis were as follows: 0 min, 80% A; 2 min, 70% A; 4 min, 40% A; 9 min, 15% A; 14 min, 10% A; 15.5–17.3 min, 5% A; 17.5–20 min, 80% A. The temperature of column was maintained at 45 °C, while the automatic sampler was set a sample size of 2 μL. Both ESI + and ESI- modes were used to collect the data. For identification and alignment, all raw data were analyzed using Analyst 1.7.3 (SCIEX, Foster City, CA, USA). Precursor and fragment mass tolerances were both set at 5 ppm (17).

### 2.4 Statistical analysis

IBM SPSS Statistics 27 and Excel 2016 were used for data processing, while the Metware bioinformatics analytic platform^1^ was used for cluster analysis and intergroup differences. A significant difference in the data is indicated by *p* < 0.05, whereas a highly significant difference is shown by *p* < 0.01.

## 3 Results and discussion

### 3.1 Effect of PSHJ on the mouse physiological state

After the modeling stage, there were notable variations in the four indicators of blood lipids (TC, TG, HDL-C, and LDL-C) between the Mol and Con group (*p* < 0.01), proving that the mouse model of hyperlipidemia was successfully established (Supplementary Table S1 and Supplementary Figure S1).

**Figure 1:**
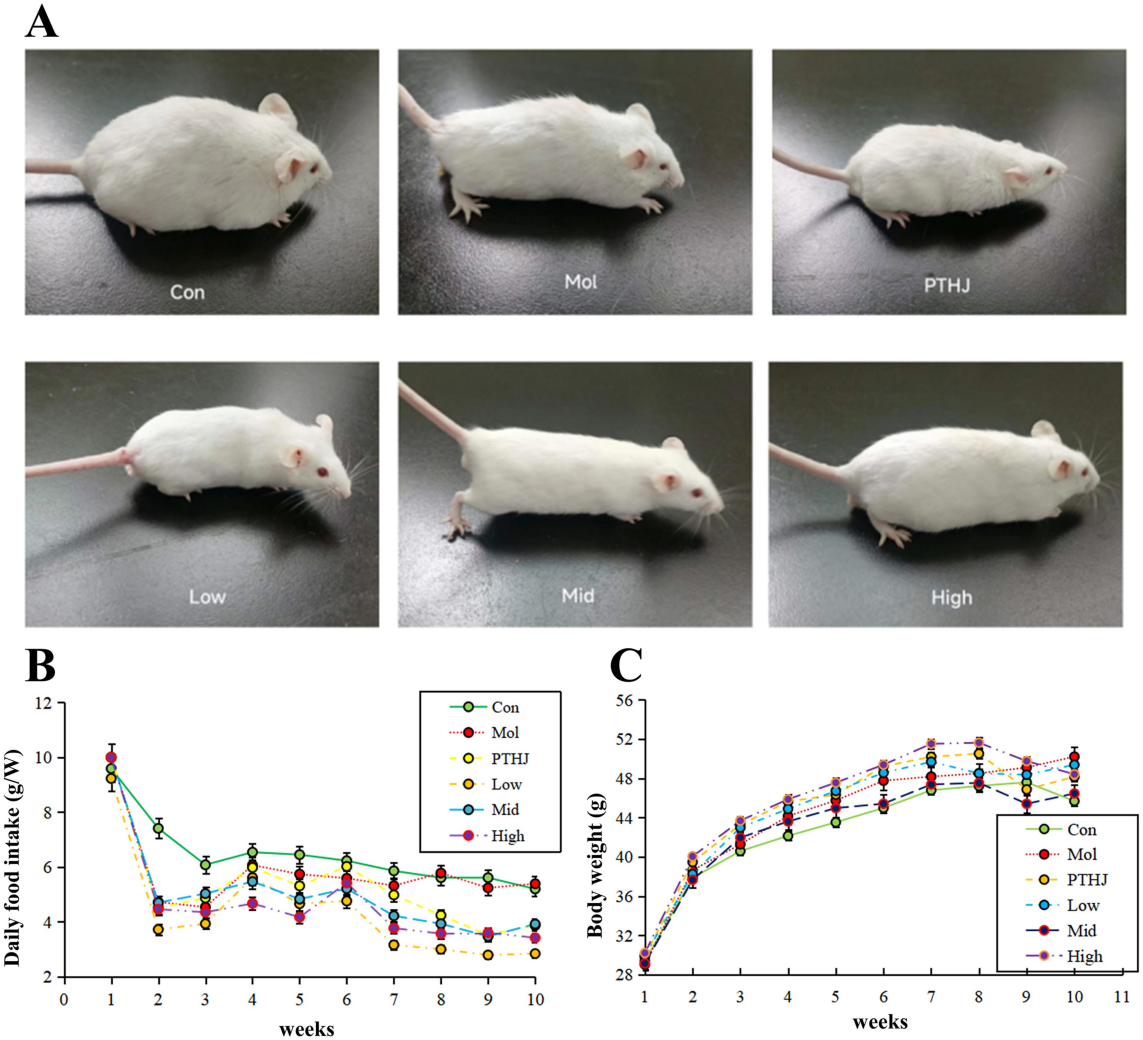
Evaluate the effect of PSHJ on the physiological condition of mice through general morphology **(A)**, daily food consumption **(B)** and body weight **(C)**. Con: normal control group; Mol: model group; PTHJ: ordinary Huangjiu group; Low: low-dose *Polygonatum sibiricum* Huangjiu group; Mid: medium dose *Polygonatum sibiricum* Huangjiu group; High: high-dose *Polygonatum sibiricum* Huangjiu group.

Before the end of the entire experiment, the state of each group of mice was evaluated (Figure 1A). The mice in Con group had smooth fur hair, clear and transparent eyeballs, dense beard growth, and normal brownish yellow feces. They were sensitive, quick to react, and had convenient and energetic movements. Mol group mice had hair loss on the tail, messy hair on the back, cloudy and dull eyes, sparse and short whiskers, slow reactions, black green feces, and slow movements. The physical and biological behavior of mice in the High and Mid groups were closest to that of the Con group. The appearance and biological behavior of PTHJ and Low group mice were similar to those of the Mol group, but their fur color slightly better and hair slightly longer. Overall, the improvement effect of PTHJ and low-dose PSHJ were basically the same, while the improvement effect of medium and high-dose PSHJ was more significant, which may be related to the content of polysaccharide, and the main ingredients in PSHJ were shown in Supplementary Table S2.

The average daily food intake of each group throughout the investigation was displayed in Supplementary Table S3 and Figure 1B. Overall, the daily food intake of mice in the Con and Mol groups remained relatively stable, while that of the treatment groups increased slowly before intervention and decreased slowly after intervention. During the administration phase (7–10 weeks), the food intake of mice in each treatment group decreased except for the Con and Mol groups. Compared with the molding stage, both have reduced by about 1.5 g per mouse. This indicated that PTHJ and PSHJ had a certain inhibiting impact on the food intake of hyperlipidemic mice, further alleviated the symptoms of hyperlipidemia.

The average body weight of each group during the experiment was displayed in Supplementary Table S4 and Figure 1C. Overall, the body weight of Con and Mol groups gradually increased and stabilized at around 45 g and 49 g m, and that of the treatment groups gradually increased before intervention and slowly decreased after intervention. During the administration phase (7–10 weeks), mice’s body weight in the Con and Mol groups remained relatively stable, but that of different treatment groups decreased. This may be related to PTHJ and PSHJ’s suppression of high-fat diet in mice.

### 3.2 Effect of PSHJ on mice’s glucose and lipid metabolism

Hyperlipidemia typically exists alongside with other metabolic illnesses related to improper lipid metabolism, such as diabetes (18), obesity (19). The study’s specific findings are displayed in Supplementary Table S5 and Supplementary Figure S2, where the concentration of fasting blood glucose (FBG) in the Mol group was 8.12, considerably higher than that of the Con group (*p* < 0.01). Prior to intervention, each group’s FGB values differed significantly from the Con group’s (*p* < 0.01), suggesting that a high-fat diet raised mice’s blood glucose levels. The FGB of PTHJ and Low group mice decreased to 7.5 and 7.36 mmol/L, respectively; the Mid group decreased to 6.7 mmol/L, while the High group showed the largest decrease, from 8.3 mmol/L decreased to 6.1 mmol/L and return that to the level of Con group (Supplementary Figures S3A,B).

The concentration of TG, TC, LDL-C, and HDL-C in each group were shown in Supplementary Table S6 and Supplementary Figure S3. Mice with hyperlipidemia have aberrant lipid metabolism. All four blood lipid indicators were significantly different in the Mol group compared to the Con group (*p* < 0.01), with TG, TC, LDL-C up-regulation and HDL-C down-regulation. Compared with the Mol group, the TG, TC, and LDL-C of mice in treatment groups were significantly down-regulated (*p* < 0.05), whereas HDL-C was dramatically up-regulated (p < 0.01). The High group showed the most significant improvement in the four indicators of blood lipids, while the PTHJ and low groups had similar improvement effects. In summary, PTHJ and different doses of PSHJ have shown improvement in glucose and lipid metabolism abnormalities in mice with hyperlipidemia, and there was a dose–response relationship with the polysaccharide content.

### 3.3 Effect of PSHJ on organ of mouse

The mouse organ index represents the ratio of organ mass to total body mass. Deviations from the normative values of this index may indicate the presence of hypertrophy, hyperplasia, or pathological atrophy of the organs (7). In this investigation, hyperlipidemia mice demonstrated metabolic abnormalities resulting from prolonged exposure to a high-fat diet. This dietary regimen led to an accelerated accumulation of lipids within the body, thereby exacerbating the burden on the liver and kidneys. Consequently, this condition resulted in notable damage and enlargement of these organs, as evidenced by increases in the indices of liver, kidney, and epididymal fat (Supplementary Table S7 and Supplementary Figure S4). Compared with the Con group, the liver index of the Mol group mice increased by 42.22%, the kidney index increased by 61.56%, and the epididymal fat index increased more than 200%. The organ index of mice in different treatment groups significant decreased compare to the Mol groups (*p* < 0.01), and it gradually decreased with the increase of PSHJ dose. Nevertheless, the comparison of liver indices between the PTHJ group and the Low group did not reveal any statistically significant differences, indicating that both groups have similar improvement effects on liver hypertrophy in hyperlipidemic mice, which may be related to their similar polysaccharide content.

**Figure 2:**
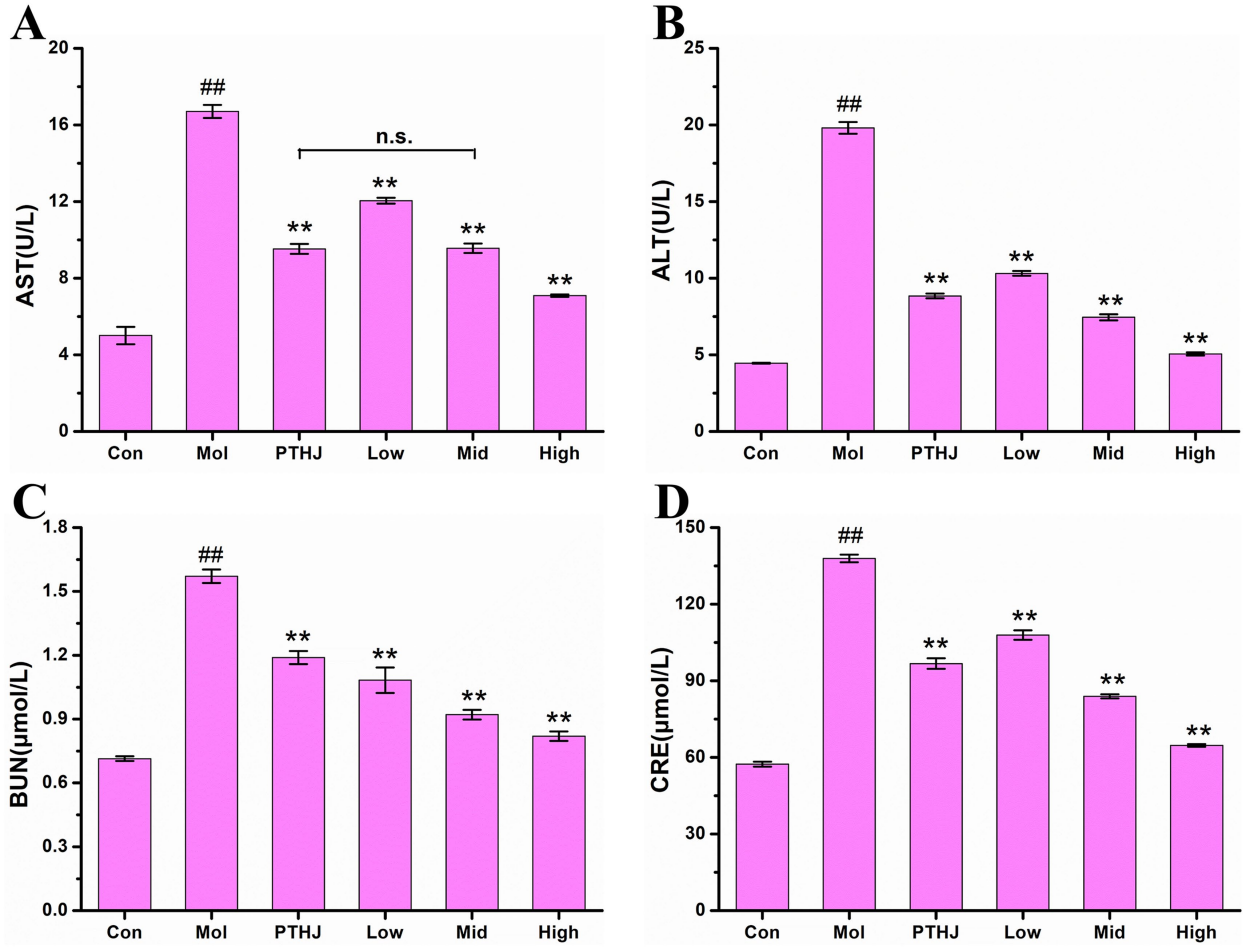
Effect of PSHJ on AST **(A)**, ALT **(B)** activity and BUN **(C)**, CRE **(D)** content in serum of hyperlipidemic mice. Con: normal control group; Mol: model group; PTHJ: ordinary Huangjiu group; Low: low-dose *Polygonatum sibiricum* Huangjiu group; Mid: medium dose *Polygonatum sibiricum* Huangjiu group; High: high-dose *Polygonatum sibiricum* Huangjiu group. ^##^*p* < 0.01 versus the normal control group, **p < 0.01 versus the model group.

The activity of aspartate aminotransferase (AST) and alanine aminotransferase (ALT) in serum are usually utilized as an indicator to evaluate liver function in clinical practice (20). The results were shown in Supplementary Table S8 and Figure 2. In comparison to the Con group, the activities of AST and ALT in Mol group exhibited a significant increase (*p* < 0.01), indicating that hyperlipidemia leads to liver damage (21). After intervention, the liver damage was relieved because the AST and ALT activities of PTHJ, Low, Mid, and High groups were significantly reduced (*p* < 0.01) compared with the Mol group. Based on the activity levels of ALT and AST, the High group exhibited the most significant protective effect against liver damage, and that of PTHJ more akin to the Low group, whereas the Mid group was similar to the High group.

Creatinine (CRE) and urea nitrogen (BUN) are often utilized as biomarkers for assessing kidney function, and their levels beyond the normal range suggest varying extents of impairment in kidney function (22). The contents of BUN and CRE in mouse serum were shown in Supplementary Table S8 and Figure 2. In comparison to the Control group, the levels of BUN and CRE in the Mol group exhibited a significant increase (*p* < 0.01), indicating that hyperlipidemia leads to kidney damage. PTHJ and PSHJ had the protective effects on kidney injury because the concentrations of BUN and CRE in PTHJ, Low, Mid, and High groups exhibited a statistically significant decrease in comparison to the Mol group (*p* < 0.01). According to BUN and CRE content, the High group showed the greatest protective effect on kidney damage, and that of PTHJ exhibited a closer alignment with the Low group, whereas the Mid group demonstrated a greater proximity to the High group.

**Figure 3:**
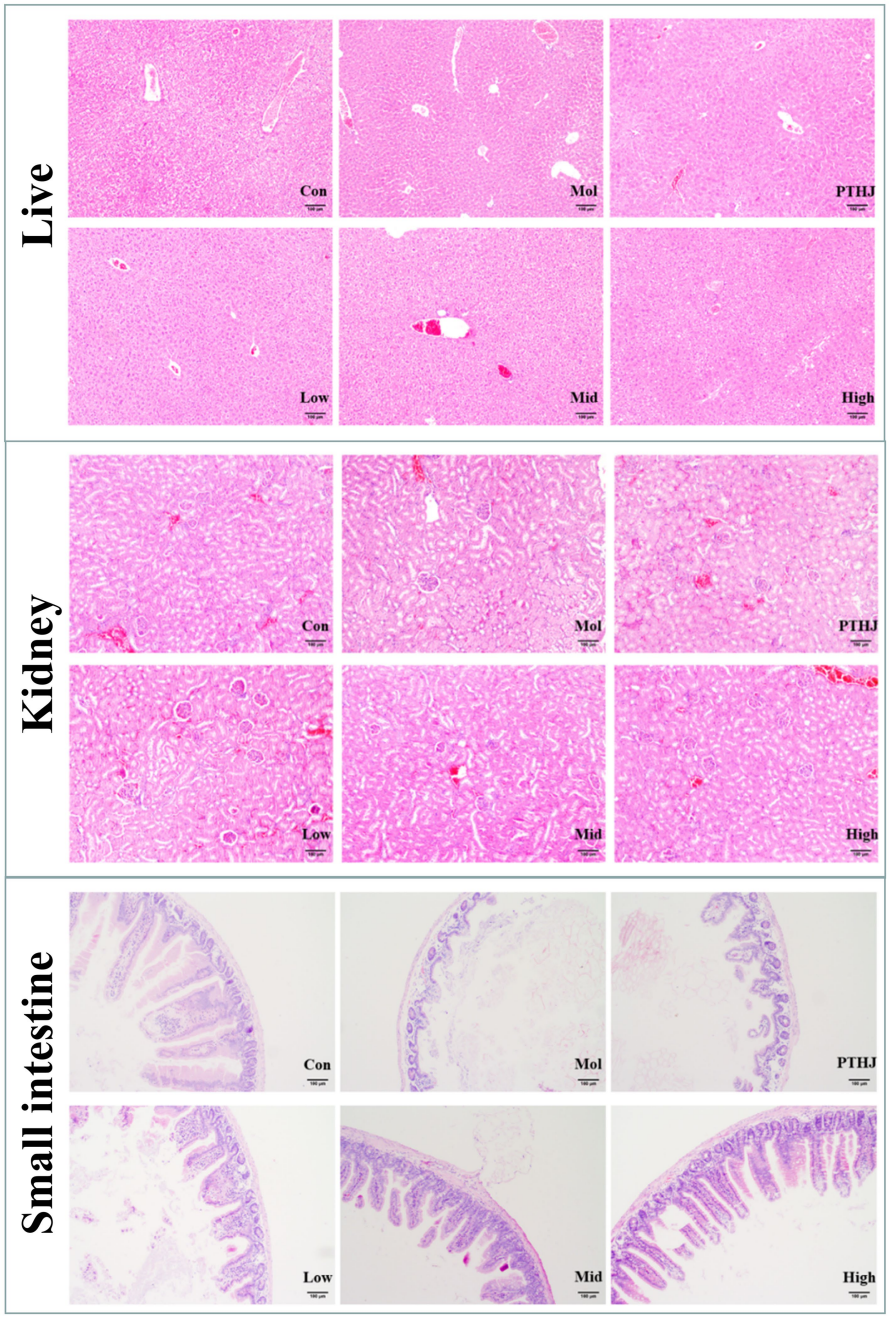
Histological staining of the liver, kidney, and small intestine of each experimental group. Con: normal control group; Mol: model group; PTHJ: ordinary Huangjiu group; Low: low-dose *Polygonatum sibiricum* Huangjiu group; Mid: medium dose *Polygonatum sibiricum* Huangjiu group; High: high-dose *Polygonatum sibiricum* Huangjiu group.

Histological staining of tissue sections from the liver, kidney, and small intestine slices was used to further investigate the protective effects of PSHJ against organ damage (Figure 3). The morphological characteristics of liver cells in the Con group were observed to be normal, exhibiting a systematic arrangement, uniform size, distinct intercellular boundaries, and the absence of lesions. Nevertheless, the liver tissue of the Mol group exhibited a significant accumulation of lipid droplets, and the cells showed compression without obvious intercellular boundaries, indicating severe cell pathology. After intervention, liver injury was alleviated in varying degrees. Among them, the High group had the least degree of liver injury, and the cell morphology was similar to the Con group, with uniform size, clear intercellular boundaries and regular arrangement, but still deposited some fat droplets. The kidney tissue cells in the control group exhibited a distinct structure, consistent morphology, and orderly distribution. The glomeruli were not dilated, and both the volume and spacing of the renal sacs were within normal limits. Conversely, the kidney tissue cells in the Mol group displayed a less defined structure, with a noticeable trend of glomerular expansion. There were fat vacuoles and fat droplets in the kidney tissue, and the glomerulus was enlarged due to excessive lipid deposition. After intervention, kidney injury was alleviated in varying degrees. The High group had shown the least degree of kidney damage, and the morphology of kidney cells exhibited similarities to that observed in the Con group. The histological examination of the small intestine tissue in the Col group revealed a normal architecture devoid of any lesions. The glands within the mucosal layer exhibited a uniform, orderly, and upright arrangement, with minimal spacing between them and the robust muscular layer of the mucosa. However, the Mol group’s small intestine mucosal glandular form demonstrated notable atrophy, with an increase in glandular spacing and a drop in number or absence. According to these findings, hyperlipidemic mice’s small intestinal mucosa thins and their small intestine tissue may sustain more injury. Conversely, when the dosage of PSHJ rose, the glandular morphology gradually recovered and in the High group, it grew closer to that of the Con group.

In summary, the assessment of biomarkers and HE staining demonstrated that PTHJ and various dosages of PSHJ exhibited a protective effect against damage to the liver, kidneys, and small intestine induced by hyperlipidemia in murine models. Furthermore, a dose–response relationship was observed in relation to their polysaccharide content.

### 3.4 Analysis of gut microbial structure

To reveal the mechanism of PSHJ in alleviating hyperlipidemia in mice, metagenomics sequencing was performed on the gut microbiota of each group of mice. More than 60,000 bacterial sequences and 30,000 fungal sequences were sequenced from the feces of each group (Supplementary Tables S9, S10). The alpha diversity dilution curves for each group exhibit a tendency to plateau as the quantity of sequencing increases (Supplementary Figure S5), indicating that the sequencing data volume were sufficient and reliable. The alpha diversity index of each group was summarized in Supplementary Tables S11, S12. The Chao index indicated a notable reduction in the diversity of gut microbiota in hyperlipidemic mice (Mol group) (23). On the other hand, PSHJ intervention seems to partially restore gut microbial diversity, and the High group has the greatest improvement.

**Figure 4:**
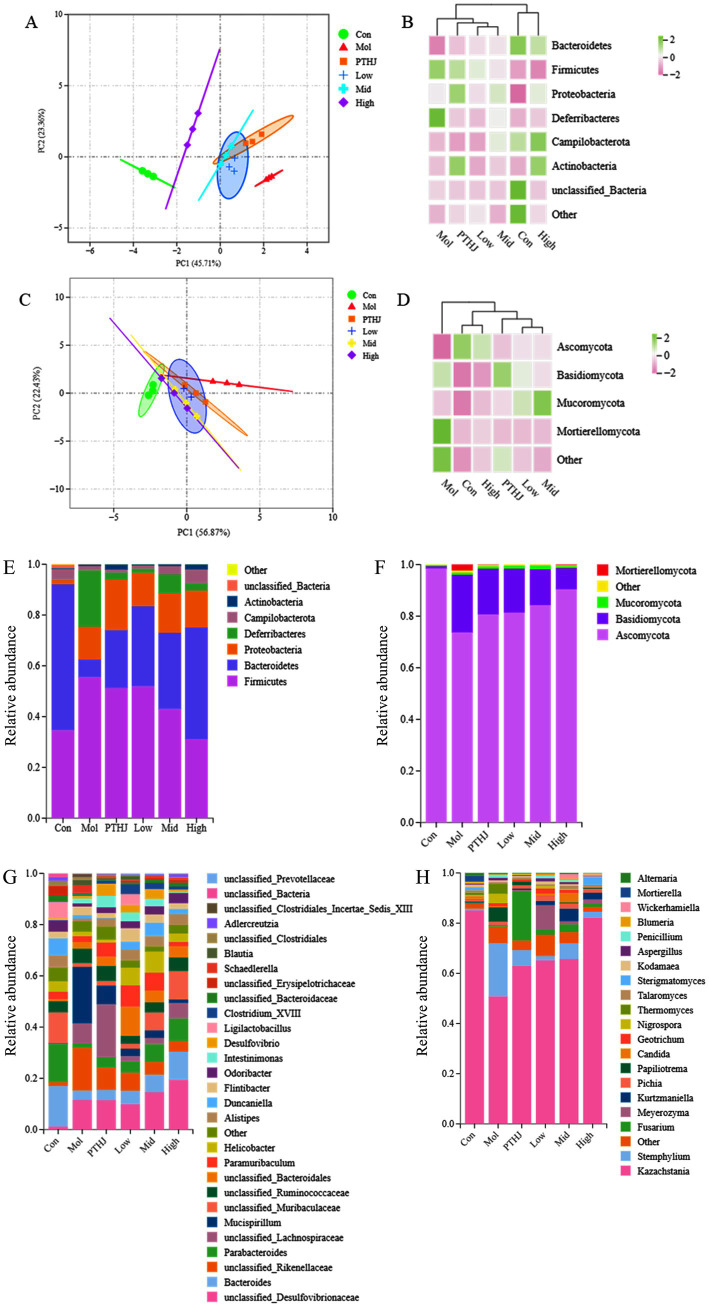
Analysis of gut microbiota composition in each group. PCA **(A,C)** and clustering heat maps **(B,D)** of each group. Species annotation of mouse gut microbiota in phylum **(E,F)** and genus **(G,H)** level. Among them, **A**,**B**,**E**,**G** represent bacteria, **C**,**D**,**F**,**H** represent fungi.

The differences in species abundance distribution between groups can be quantified through statistical distance analysis. The distance between groups was calculated using Bray-Curtis and UnFrac statistical algorithms (Supplementary Tables S13, S14). The findings demonstrated that the Mol group and the Con group had the most differences in gut microbiota structure., and the difference gradually decreased after intervention. This conclusion had also been confirmed by principal component analysis (PCA) and cluster analysis (Figures 4A,C). Following the PSHJ intervention, the High group’s microbial structures were the most similar to those of the Con group, indicating that a high dose of PSHJ is most effective in reestablishing the gut microbiota. In cluster analysis, the distribution of colors can reveal the degree of similarity between groups. In bacterial clustering heat map (Figure 4B), Con was grouped with High, Low was grouped with Mid, PTHJ was grouped with Low and Mid. And fungal clustering analysis was consistent with that of bacterial (Figure 4D). The results indicated that gut microbiota structure in mice is altered by hyperlipidemia, but PTHJ and PSHJ could reduce this difference.

The species annotations at genus and species level of mouse gut microbiota were shown in Figures 4E–H. In bacterial annotation, the Mol group showed the greatest difference with the Con group, the proportion of *Firmicutes* increased from 34.67 to 55.58%, while that of *Bacteroidetes* decreased from 57.48 to 6.89%. Meanwhile, the proportions of *Deferribacteres* and *Proteobacteria* increased to 22.23 and 12.91%, respectively. After intervention, the proportion of *Bacteroidete*s was increased and the growth of *Firmicutes* was inhibited. The High group showed the most significant changes, and the proportions of the two were similar to that of the Con group. In the fungal annotation, *Ascomycota* was the dominant fungus in each group, with the highest proportion of 98.51% in the Con group, decreased to 73.7% in the Mol group. However, its proportion increased in different treatment groups. The total amount of *Bacteroidetes*, *Firmicutes*, *Proteobacteria*, and *Actinobacteria* accounted for 98% of the total gut microbiota, and they play important roles in various physiological and metabolic activities of the colon in the body (24). Some studies have found an increase in the abundance of *Firmicutes* and a decrease of *Bacteroidetes* in hyperlipidemic mice (25). Therefore, *Bacteroidetes* and *Firmicutes* were, respectively, referred to as lean bacteria and fat bacteria (26). Meanwhile, the highest abundance of *Ascomycota* in the Con group indicated its potential role in regulating lipid metabolism.

*Bacteroides* with an abundance of 15.85% in the Con group was decreased to 3.78% in the Mol group, and then it increased in the treatment groups compared to the Mol group. At the same time, the proportion of *Parabacterioides*, *Alistipes*, *Odorobacter*, and *unclassified*_*Muribaculaceae*, *Erysipelotrichaceae* in the treatment group also increased. Research had shown that *Parabacterioides* and *Odoribacter* could produce SCFAs and cholic acid, which are crucial regulators of the body’s lipid balance (27). In this study, *Mucispirillum* abundance rose in the Mol group while falling in the Con and treatment groups., which was similar to previous research findings (28). According to a different study, *unclassified Muribaculaceae* is crucial to the breakdown of complex polysaccharides (29). It was the dominant bacterium in Con group (12.03%), its proportion decreased to 1.5% in the Mol group and rebounded in the treatment group.

PTHJ and PSHJ have a regulatory influence on the disruption of gut microbiota in mice caused by hyperlipidemia, and indirectly regulated blood lipids through the enhancement of the gut microbiota composition (30).

### 3.5 Analysis of the relationship between gut microbiota and SCFAs in mice

Among the various SCFAs, acetic acid, propionic acid, and butyric acid account for around 90% of the total. They have an important influence on the metabolic processes of organs and tissues such as the intestine and liver, as well as playing an essential regulatory function in cholesterol synthesis and lipid metabolism (31). Eight SCFAs’ concentration in mouse feces were displayed in Supplementary Table S15, and the comparative analysis of four important types in each group was shown in Supplementary Figure S6. The amount of acetic acid, propionic acid, butyric acid, and valeric acid in Mol group mice was considerably lower than that in the Con group (*p* < 0.01), with reduced by 70.0, 86.4, 99, and 85.9%, respectively. It may be due to hyperlipidemia disrupted the gut microbiota of Mol group mice, which resulted in fewer functional bacteria that produce SCFAs (32). In comparison to the Mol group, the levels of acetic acid, propionic acid, butyric acid, and valeric acid in the treatment groups of mice were significantly elevated (p < 0.01), and they increased with the increase of PSHJ dose. Among them, High group showed the largest increase in the content of these four types of SCFs. Nevertheless, the analysis revealed no statistically significant differences in the concentrations of acetic acid, propionic acid, and valeric acid between the PTHJ group and the Low group of mice. It may be related to the content of polysaccharides, which as prebiotics could promote the growth and reproduction of SCFAs producing functional bacteria and indirectly increase the content of SCFAs.

**Figure 5:**
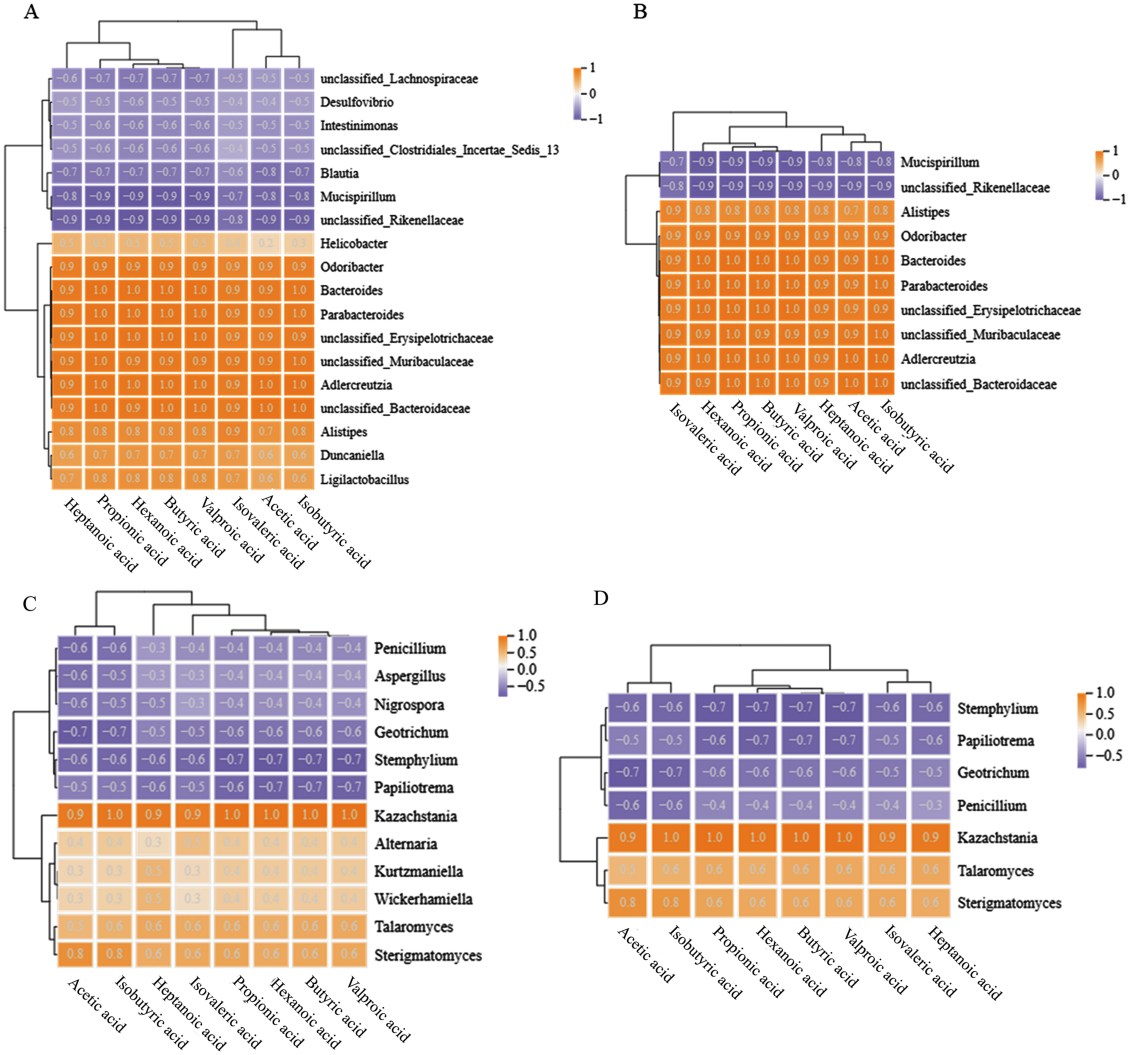
Correlation analysis between gut microbiota and SCFAs in mice at the genus level. **(A,B)** Represent bacteria, **(C,D)** represent fungi; **(A,C)** show all objects with correlations, while **(B,D)** show objects with correlations higher than 0.8 and 0.6, respectively.

The correlation analysis between mouse gut microbiota (bacteria, fungi) and SCFAs was shown in Figure 5. A negative correlation coefficient indicates a negative correlation, a positive correlation coefficient indicates a positive correlation, and a larger absolute value indicates a higher correlation.

There were a total of 8 bacterial genera in the mouse gut that have a positive correlation of 0.8 or higher with SCFAs, all of which were dominant genera in the Con and treatment groups, including *Parabacterioides*, *Bacteroides*, *Odoribacter*, etc. In addition, the dominant bacteria *Mucispirillum* and *unclassified_Rikenellaceae* in the Mol group showed a negative correlation with SCFAs. It is worth noting that some bacterial genera were completely positively correlated with SCFAs, such as *unclassified_Bacteroidaceae* and *Adlercreutzia*, which have a positive correlation coefficient of 1 with acetic acid, isobutyric acid, butyric acid, valeric acid, and propionic acid. Meanwhile, the dominant fungi *Sterigmatomyces* and *Kazakhstania* in the Con and treatment groups demonstrated a positive correlation with SCFAs greater than 0.8, and *Kazakhstania* was completely positively correlated with valeric acid, butyric acid, hexanoic acid, propionic acid, and isovaleric acid. The genera of *Stemphalium*, *Papiliotrema*, *Geotrichum*, and *Penicillium* showed a negative correlation with SCFAs greater than 0.6, which were the dominant fungi in the Mol group mice.

In summary, mice with hyperlipidemia have disturbed gut microbiota, which results a decrease in the abundance of SCFAs producing functional bacteria such as *Parabacterioides*, *Bacteroides*, *Odoribate*, and *Kazachstania*, indirectly resulting in a downregulation of SCFAs levels. Intervention with PTHJ and different doses of PSHJ promotes the growth of these microorganisms and helps to restore normal SCFAs levels in mice. Due to SCFAs play a crucial part in controlling lipid metabolism (33), we speculate that the improvement of hyperlipidemia in mice by PTHJ and PSHJ was related to their regulation of the gut microbiota.

### 3.6 Widespread targeted lipidomics analysis of mice

A total of 46 subtypes of lipids were found in each mouse group’s fecal samples (Supplementary Figure S7), including 958 lipid compounds, belonging to six primary lipids: sterol esters (SE), sphingolipids (SP), fatty acyls (FA), glycerophospholipids (GP), glycerolipids (GL), and Prenol lipids (PR). They accounted for 2.29, 11.17, 31.12, 16.7, 38.11, and 0.31% of the total lipids, respectively. GP, SP, and GL were the main lipids in mouse feces, and GL composed of triacylglycerol (TG) and diacylglycerol (DG) is also known as fat. TG are the main evaluation indicators of hyperlipidemia. Therefore, analyzing the differences and changes of GL among different groups of mice can help explore the pathological mechanism of hyperlipidemia at the microscopic level.

**Figure 6:**
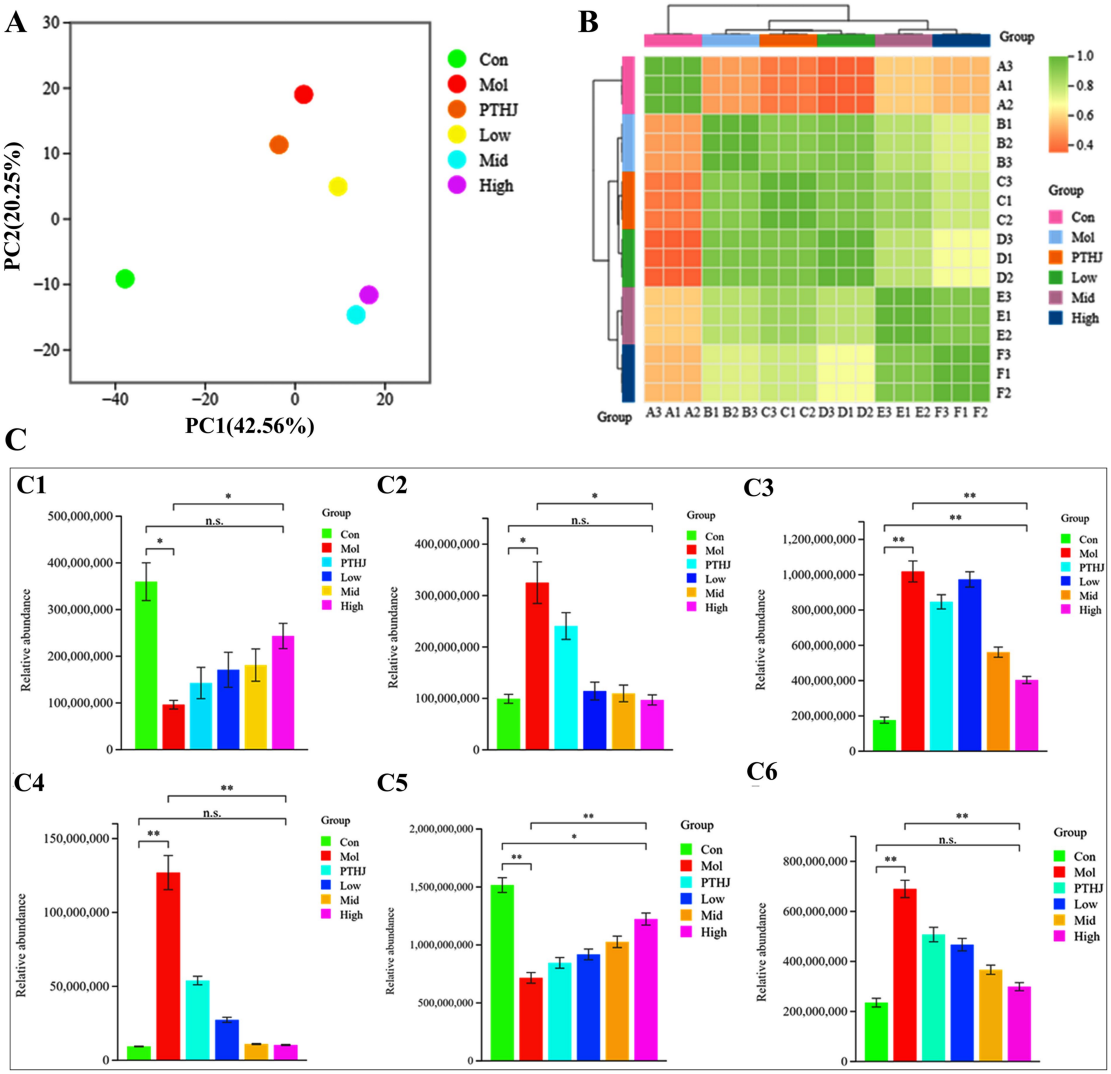
Overall differences in lipid compounds among different groups of mice. **(A)** PCA; **(B)** clustering heat maps; **(C)** Relative abundance of primary lipids. C1, C2, C3, C4, C5, C6 represent primary lipids FA, GL, GP, PR, SP, ST, respectively.

PCA analysis effectively demonstrated the inter group differences of lipid compounds, which were completely separated in PCA diagram (Figure 6A). Compared with the Mol group, the distance between Con, Mid, and High groups was relatively long, resulting in significant differences in their lipid composition. Meanwhile, PTHJ and Low group had short distances and similar lipid compositions with Mol group. The correlation clustering analysis confirmed conclusion, with PTHJ and Low being clustered together, Mid being clustered together with High, and Con forming its own category (Figure 6B). The groups of PTHJ, Low, Mid, and High produced many differential lipids compared to Mol group, which mainly belong to GL, GP, and SP. Compared with Con group, the Mol group’s GL abundance was significantly up-regulated (*p* < 0.05), but regressed in different treatment groups (Figure 6C). In addition, the abundance of the other five types lipids in Mol group revealed notable variations from the Con group (*p* < 0.05), and this difference was reduced in treatment groups. It was worth noting that as the dosage of polysaccharides in the treatment group increases, the abundance of lipids becomes closer to that of the control group, indicating a dose–response relationship.

The lipid composition of organisms is influenced by various factors, significant differences in lipids between groups are usually representative. Took Con, Mol, and High group as representatives, and selected the top 10 difference multiples lipids in terms of their intergroup as the key differential lipids, as shown in Supplemntary Figure S8. Compared with the Con, the highest upregulated lipid in Mol was TG, with the largest increase in TG (18:1_18:2/20:1) and TG (12:0_14:0_16–0), and DG and carnitine (CAR) upregulated, sphingomyelin (SM) significantly downregulated, etc. Compared with Mol group, the downregulated differential lipids in High group mainly were TG and DG, including TG (18:1_18:2/20:4), TG (15:0_18:2_18:2), TG (16:0_16:2:20:5), etc. In addition, ceramide (Cer) and CAR were also significantly downregulated. Further differential analysis of secondary lipid abundance in Con, Mol, and High groups was shown in Supplementary Figure S9. TG is a secondary lipid in GL, it is one of the main forms of energy storage in the body, but high levels of TG are linekd to myocardial infarction, coronary artery disease, hypertension, and hyperglycemia (34). DG belongs to GL, which is an important component of the cell membrane in addition to energy storage, but high levels of DG are related to cardiovascular disease (35), fatty liver (36), and insulin resistance (37). The abundance of TG and DG in the Con and High groups were significantly downregulated compared to Mol group (*p* < 0.01), but there was no substantial distinction between the Con and High groups. The high-fat diet led to excessive fat intake in the Mol group mice, resulting in a significant increase in their abundance. However, this trend was inhibited after high-dose PSHJ intervention. At the same time, serum TG content in the Mol group was also far higher than that in the Con group and treatment groups. This result further proves that the intervention of PSHJ promotes the catabolism of TG and DG.

Mice in the Mol group had substantially more Phosphatidylethanolamine (PE) than those in the Con and High groups. (*p* < 0.01). PE is an important phospholipid lipid in cell membranes, but liver and cardiovascular diseases may be related to abnormal PE metabolism (38). The abnormal increase in PE abundance in Mol group mice may be due to lipid metabolism regulation in response to organ damage caused by hyperlipidemia. Furthermore, in contrast to the Con and High groups, the phosphatidylcholine (PC) abundance in the Mol group mice was significantly reduced (*p* < 0.01). PC is also an important component of the cell membrane and will be decreased in rats fed with HFD (39), which were similar to that of the Mol group. After high-dose PSHJ intervention, the abundance of PC significantly increased (*p* < 0.01). High levels of SM are related to coronary heart disease (40). The intervention of high-dose PSHJ significantly reduced the abundance of SM in mice (*p* < 0.01) and reduced coronary heart disease risk.

In short, PSHJ had a regulatory effect on key differential lipids such as TG, DG, SM, and PE, thus relieving hyperlipidemia in mice. However, the specific regulatory pathway needs further analysis.

### 3.7 Analysis of the KEGG pathway for differential lipids in different comparison groups

**Figure 7:**
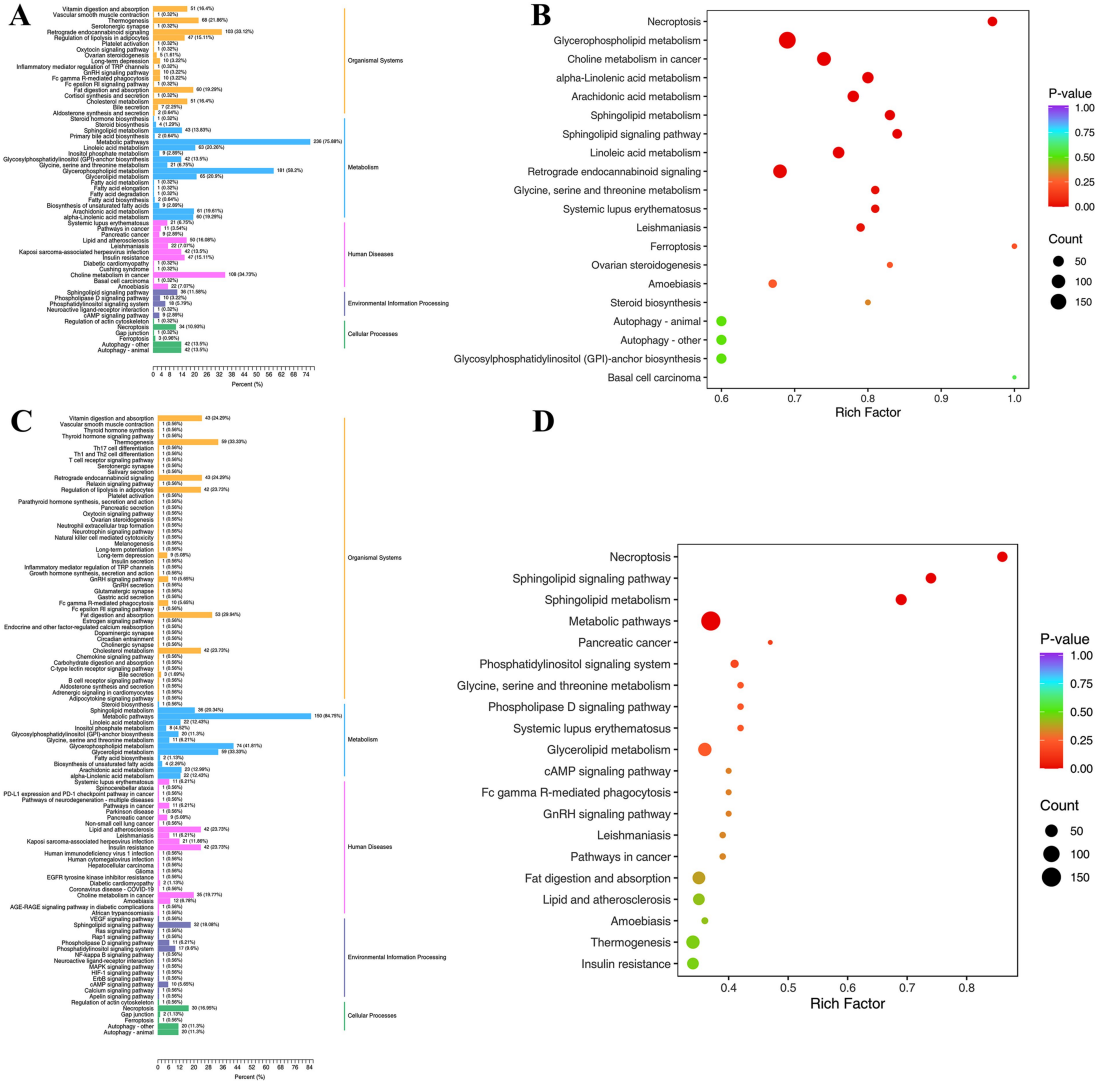
Differential lipid KEGG classification **(A,C)** and enrichment analysis **(B,D)** of comparative groups. **(A,B)** Represent Con vs. Mol group; **(C,D)** represent High vs. Mol group.

Mapping lipidomics data to Kyoto Encyclopedia of Genes and Genomes (KEGG) metabolic pathways can identify the metabolic pathways impacted by particular biological situations or disease states (41). As shown in Figure 7, the KEGG pathways annotated by differential lipids in different comparison groups were classified and enriched for analysis. The annotated KEGG pathways include five major modules, such as organismal systems, metabolism, human diseases, environmental information processing, and cellular processes. Each module contains more than ten to dozens of KEGG secondary pathways. In the two comparison groups (Con vs. Mol, Mol vs. High), many KEGG pathways were annotated in the metabolic and organismal systems, so a focused analysis was conducted on the metabolic module and its secondary KEGG pathway.

The metabolic pathways account for the highest proportion in the Con vs. Mol metabolism module (75.88%), followed by glycerophospholipid metabolism (58.2%), while glycerolipid metabolism (20.9%) and linoleic acid metabolism (20.26%) both account for over 20%. The proportion of metabolic pathways in Mol vs. High increased to 84.75%, glycerophospholipid metabolism decreased to 41.81%, while glycerolipid metabolism increased to 33.33% and linoleic acid metabolism decreased to 12.43%. Enrichment analysis confirmed the he above result, the KEGG pathway of Con vs. Mol was significantly enriched in glycerophospholipids, linoleic acid, and alpha linoleic acid metabolism, while sphingolipid metabolism and sphingolipid signaling pathway were significantly enriched in Mol vs. High, and no accumulation of linoleic acid metabolism. The proportion increase of metabolic pathway and glycerolipid metabolism in Mol vs. High suggested that high-dose PSHJ promotes the catabolism of GL through this pathway, achieving the effect of alleviating hyperlipidemia in mice. This was consistent with the decrease in GL abundance in mice after high-dose PSHJ intervention. In addition, linoleic acid as an essential polyunsaturated fatty acid is not only a crucial part of the cell membrane, but also has physiological functions such as regulating inflammation, blood pressure, and immune response in the body. The proportion decrease of linoleic acid metabolism pathway in Mol vs. High suggested that high-dose PSHJ could slow down the breakdown of linoleic acid lipids in mice and regulate inflammation caused by hyperlipidemia.

### 3.8 Mechanism of PSHJ in relieving hyperlipidemia in mice

**Figure 8:**
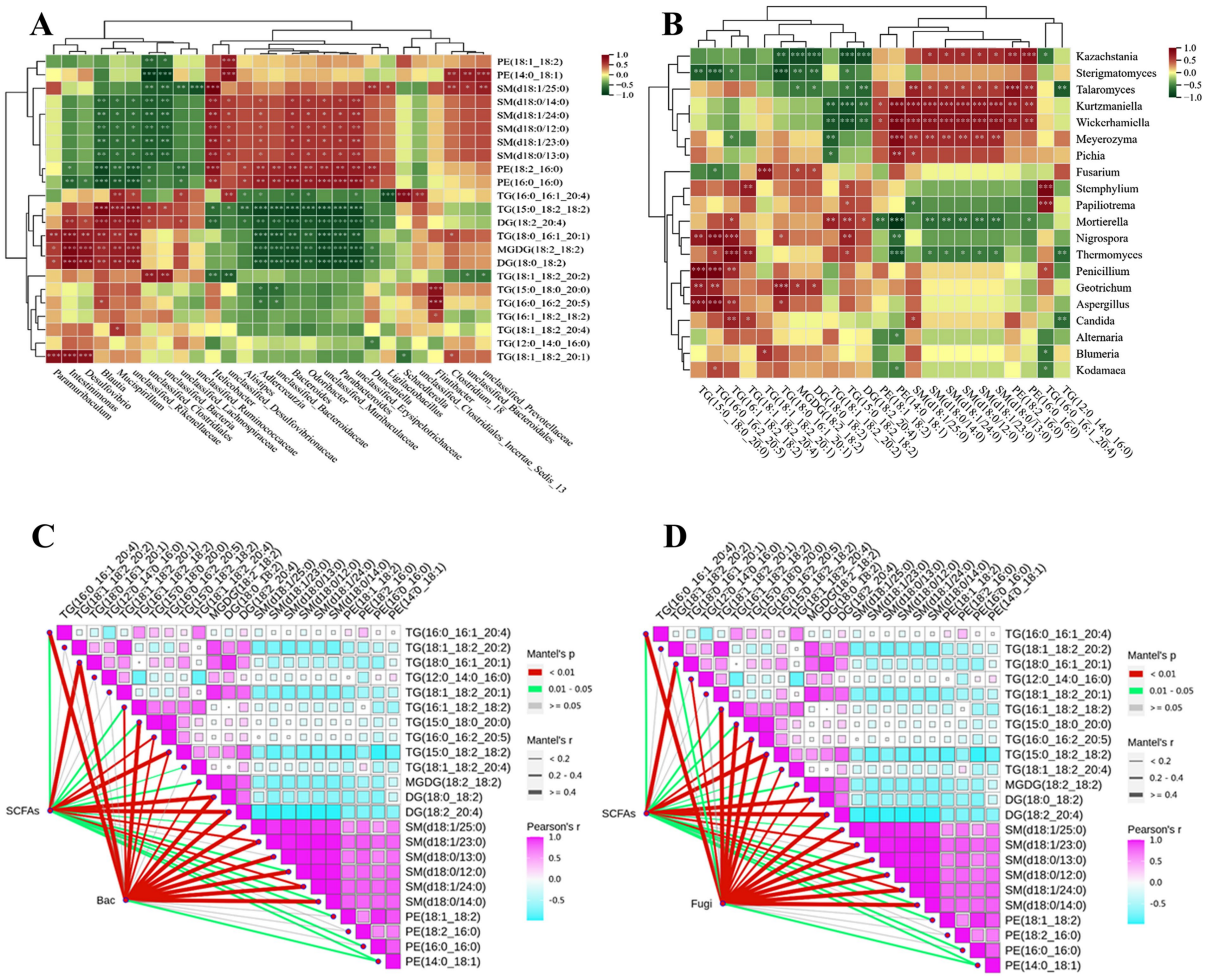
Correlation analysis of key lipids with mouse gut bacteria **(A)**, fungi **(B)**. Correlation analysis of key lipids with short chain fatty acids and mouse gut bacteria **(C)**, fungi **(D)**.

Gut microbes affect the lipid metabolism of the host through various mechanisms, analyzing the correlation between key differential lipids and gut microbiota can help explore the mechanism of PSHJ alleviating hyperlipidemia in mice. As shown in Figure 8A, TG and DG showed an extremely significant negative correlation with dominant bacteria such as *Odorobacter*, *Parabacterioides*, *unclassified_Erysipelotrichaceae* in Con and treatment groups. The TG content in Con and High group mice was far less than that of the Mol group, suggesting that the above bacteria usually appear in low abundance TG and DG environments. The dominant bacteria of Mol group such as *Mucispirilum*, *unclassified_Rikenellaceae*, and *unclassified_Lachnospiraceae* exhibited a positive association with TG and DG, and TG in the Mol group was significantly upregulated. In addition, key differential lipids such as PE and SM also exhibited similar phenomena, showing a highly significant positive correlation with bacteria such as *Odorobacter* and *Parabacterioides*, and a highly significant negative association with *Mucispirillum*, *unclassified_Rikenellaceae*, and others. At the fungal level (Figure 8B), TG, DG showed a significant negative correlation with dominant fungi of Con and treatment groups such as *Kazachstania* and *Sterigmatomyces*, while showing an obvious positive connection with dominant fungi of Mol group such as *Thermomyces*, *Penicillium*, and *Aspergillus*. In addition, PE and SM showed significant positive correlations with *Kazachstania*, *Kurtzmaniella*, *Wickerhamiella*, and significant negative correlations with *Thermomyces* and *Mortierella*. In summary, there was a correlation between the abundance of key differential lipids and the dominant microorganisms in each group of mice, which may be the mechanism for regulating lipid metabolism in hyperlipidemic mice.

We had found the dominant bacteria and fungi such as *Odorobacter*, *Parabacterioides*, and *Kazachstania* in Con and treatment groups showed significant positive correlations with SCFAs, while they also showed significant negative correlations with TG and DG. Therefore, a correlation analysis was conducted between gut microbiota, SCFAs, and key differentially lipids, as shown in Figures 8C,D. The interaction nodes represent the correlations between SCFAs, bacteria (Bac) and fungi (Fugi) with key differentially lipids, and the three showed extremely significant correlations at certain nodes, such as SCFAs, Bac, and TG (18:1_18:2:20:2), TG (16:0_16:2:20:5), TG (15:0_18:2_18:2), DG (18:0_18:2), DG (18:2:20:4), SM (d18:1/23:0). Meanwhile, SCFAs, Fugi, and key differential lipids such as TG (16:0_16:1_20:4), TG (18:1_18:2:20:2), DG (18:0_18:2), DG (18:2:20:4), SM (d18:1/24:0), SM (d18:0/12:0), PE (14:0_18:1) also showed significant correlations.

To sum up, it was speculated that the mechanism by which PTHJ and PSHJ alleviated hyperlipidemia in mice was that PTHJ and PSHJ provided polysaccharides, which, as prebiotics, improved gut microbiota diversity. Then it facilitated the proliferation of SCFAs-producing functional bacteria such as *Odorobacter*, *Parabacterioides*, and *Kazakhstania*, indirectly increasing SCFAs levels and forming a virtuous cycle. Then, SCFAs and polysaccharides acted on the metabolic pathways of glycerophospholipids, glycerols, and fatty acids in some way, promoting the breakdown metabolism of TG and DG, thereby alleviating hyperlipidemia in mice. However, there are still many aspects of this study that need to be further investigated, such as the isolation and characterization of effective ingredients in PSHJ that alleviate hyperlipidemia, investigation of the structure–function relationship of the active constituents, and the direct functional verification of PSHJ, microbial communities, SCFAs and lipid metabolism correlation through methods such as microbial depletion or SCFAs inhibition studies.

## 4 Conclusion

The findings of this study indicate that PSHJ may have beneficial effects in mitigating hyperlipidemia in mice induced by HFD. These effects include the suppression of appetite and organ damage (liver, kidney, and small intestine), reduction of body weight and blood sugar growth, decrease of serum levels of TC, TG, and LDL-C, and elevation of HDL-C levels. The relief impact of PSHJ on hyperlipidemia in murine models shown a dose–response relationship in polysaccharide concentration. Through the combination of microbiology and extensive targeted lipidomics, it was found that HFD-induced hyperlipidemia leads to disrupted gut microbiota structure, decreased microbial diversity and SCFAs content, and abnormal lipid metabolism. After intervention with different doses of PSHJ, the diversity of gut microbiota, and abnormal lipid metabolism were restored, and the content of SCFAs was significantly increased. The improvement effect was dose-dependent on the polysaccharide content of Huangjiu. A notable association was observed among SCFAs, gut microbiota and key differential lipids. The mechanism by which PSHJ alleviates hyperlipidemia in mice may be that PTHJ and PSHJ provided polysaccharides as prebiotics to hyperlipidemic mice, enhancing the diversity of gut microbiota and promoting the growth of SCFAs-producing functional bacteria such as *Odorobacter*, *Parabacterioides*, and *Kazachstania*, indirectly increasing SCFAs levels and forming a virtuous cycle. Then, SCFAs and polysaccharides regulated the metabolism of glycerophospholipids, glycerols, and fatty acids, promoting the breakdown metabolism of TG and DG in hyperlipidemic mice, thereby alleviating hyperlipidemia in mice.

## Data Availability

The original contributions presented in the study are included in the article/Supplementary material, further inquiries can be directed to the corresponding authors.

## Footnotes

1
https://cloud.metware.cn
