# Supplementary material for: Multi-omics techniques revealing the mechanism of Polygonatum sibiricum Huangjiu in alleviating hyperlipidemia in mice
Source: Front Nutr. 2025 Nov 27;12:1705061. doi: 10.3389/fnut.2025.1705061 (PMC12695534; doi:10.3389/fnut.2025.1705061)
Supplement: Supplementary file 1 [file Data_Sheet_1.docx]

***Supplementary Material***

**Multi-omics techniques revealing the mechanism of *Polygonatum sibiricum* Huangjiu in alleviating hyperlipidemia in mice**

Jingzhang Geng^1,2^, Guotai Zhang^1,3^, Jianwei Dong^1^*, Honglei Tian^1^*, Zaixiang Lou^4^

1.Shaanxi Province Key Laboratory of Bio-Resources, QinLing-Bashan Mountains Bioresources Comprehensive Development C. I. C., Qinba State Key Laboratory of Biological Resources and Ecological Environment, Shaanxi University of Technology, 1 East 1st Ring Road, Hanzhong 723001, China; gengjingzhang@163.com (J.G.); zshanshan2024@163.com (G.Z.); thl0993@sina.com (H.T.)

2.School of Life Science and Technology, Xi’an Jiaotong University, Xianning West Road, Xi’an 710049, China; gengjingzhang@163.com (J.G.)

3. College of Food and Chemical Engineering, Liuzhou Institute of Technology, No. 99, Xinliu Avenue, Yufeng District, Liuzhou, 545616, China; zshanshan2024@163.com (G.Z.)

4. School of Food Science and Technology, Jiangnan University, No. 1800, lihu avenue, wuxi, 214122, China; [louzaixiang@126.com](mailto:louzaixiang@126.com) (Z.L.)

* Corresponding authors: [jwdong@snut.edu.cn](mailto:jwdong@snut.edu.cn) (J.D); [thl0993@sina.com](mailto:thl0993@sina.com) (H.T.)

**Table of Contents**

| **Subject** | **Page** |
| --- | --- |
| Materials and methods | S-3 |
| Table S1 Levels of TC, TG, HDL-C, and LDL-C in the serum of Con and Mol group mice | S-6 |
| Figure S1 Comparison of serum TC(A), TG(B), LDL-C(C), and HDL-C(D) content between of blank and model group mice | S-6 |
| **The main ingredients of PSHJ** | S-7 |
| **Table S2** The main ingredients of PSHJ | S-7 |
| **Table S3** Average daily food intake of each group of mice | S-7 |
| **Table S4** Average body weight of each group of mice | S-7 |
| **Table S5** Average FGB values of each group of mice | S-8 |
| **Figure S2** FGB values of each group of mice at the sixth week | S-8 |
| Table S6 Serum TC, TG, LDL-C, and HDL-C in each group of mice | S-8 |
| Figure S3 Effect of PSHJ on glucose and lipid metabolism of mouse | S-9 |
| **Table S7** Average organ index of each group of mice | S-10 |
| Figure S4 Comparative analysis of the significance of organ index in each group of mice | S-10 |
| **Table S8** Effect of PSHJ on liver and kidney function indicators of hyperlipidemic mice | S-11 |
| Table S9 Sample sequencing quantity statistics (bacteria) | S-11 |
| Table S10 Sample sequencing quantity statistics (fungi) | S-11 |
| Figure S5 Alpha diversity dilution curve of gut bacteria (A) and fungi (B) in each group of mice. | S-12 |
| Table S11 Alpha diversity index of different samples (bacteria) | S-12 |
| Table S12 Alpha diversity index of different samples (fungi) | S-12 |
| Table S13 Bray distance matrix between different sample groups (bacteria) | S-13 |
| Table S14 Bray distance matrix between different sample groups (fungi) | S-13 |
| Table S15 Content of SCFAs in feces of different groups of mice | S-13 |
| Figure S6 Comparative analysis of SCFAs content in each group of mice | S-14 |
| **Figure S7** Lipid subclass ring diagram | S-14 |
| **Figure S8** Ranking of the Top 10 secondary differential lipids with multiple differences between mouse groups. | S-15 |
| **Figure S9** Comparison of relative abundance of representative differential lipids among different groups of mice. | S-16 |
| **Reference** | S-17 |

**Materials and methods**

1. **Chemicals**

*Polygonatum sibiricum* Huangjiu (PSHJ) and ordinary Huangjiu (PTHJ) were prepared in our own laboratory. Edible alcohol was acquired from a local supermarket. Detection kits for TC, TG, LDL-C, HDL-C, glutamic oxaloacetic transaminase (AST), glutamic-pyruvic transaminase (ALT), serum creatinine (Scr), and blood urea nitrogen (BUN) were obtained from Nanjing Jiancheng Biotechnology Co., Ltd., located in Nanjing, China. Hematoxylin, eosin and polyformaldehyde were sourced from Wuhan Servicebio Technology Co., Ltd., based in Wuhan, China. Chromatographic mobile phases of methanol, acetonitrile, isopropanol, formic acid, ammonium formate were purchased from Thermo Fisher Scientific Inc., located in Waltham, MA, USA, and all chemical reagents are HPLC grade. Potassium dichromate, phenol, potassium sodium tartrate, and sodium sulfite were acquired from Tianjin Damao Chemical Reagent Factory in Tianjin, China, and all chemical reagents are analytical grade. The standard basic and high-fat diets utilized in the animal experiments as well as the reference standard of short-chain fatty acids (SCFAs) were obtained from Xi'an Botian Biotechnology Co., Ltd., located in Xi’an, China. In addition to a variety of complex vitamins and trace minerals, the main components of the basic diet were corn, soybean flour, bran, fish powder, and chicken powder. The high-fat diet, on the other hand, contained 49% base plastic, 12% casein, 10% lard, 3% sesame oil, 20% fructose, 2% premix, 2% dicalcium phosphate, 0.5% sodium gallate, and 1.5% cholesterol.

1. **Monitoring of fundamental physiological indicators**

Careful observations were taken every day for the length of the experiment to evaluate the mice's morphology, activity, and fecal condition. Mice's morphology mostly consists of their mental state, reaction to outside stimuli, and fur color and smoothness. Color and form are the primary indicators of fecal status. In order to track changes over time, weekly body weight measurements were performed in addition to daily food intake data.

**3. Measurement of biochemical indexes**

**3.1** **Determination of fasting blood glucose levels**

A blood glucose meter (EA-11, Sinocare Biosensing Co., Ltd., Changsha, China) was used to test the fasting blood glucose (FBG) levels after each mouse had its tail blood sampled after a 12-hour fast. Before the experimental intervention started, the initial FBG levels of each mouse group were noted. The FBG levels of every mouse group were then assessed on the seventh day of every week throughout the duration of the intervention.

**3.2 Determination of blood lipid levels**

Blood samples were drawn from the mice's eye sockets into 10 mL centrifuge tubes after a 12-hour fast. These samples were then stored at room temperature for four hours. To separate the serum, the blood samples were centrifuged for 15 minutes at 4°C at a speed of 4000 revolutions per minute (r/min). The serum's concentrations of TC, TG, HDL-C, and LDL-C were then determined using commercially available assay kits. Blood lipid levels need to be measured both after the modeling of hyperlipidemia and the entire experiment.

**3.3** **Evaluation of the liver, kidney, and epididymal fat indices**

Cervical dislocation was employed for execution after anesthesia. The mice were then dissected right away, and tweezers were used to carefully remove the liver, kidneys, and epididymal fat. Following extraction, the tissues were gently washed with physiological saline solution, and filter paper was used to remove any remaining surface moisture. After that, the tissues were measured and weighed. Formula (1) was used to determine the liver, kidney, and epididymal fat indices.

Organ and epididymal fat Index/% =$\frac{Organ and epididymal fat weight/g}{Body weight/g}$ × 100% (1)

**3.4** **Assessment of the liver and kidney function**

Serum levels of ALT, AST, Scr, and BUN were measured using assay kits in order to evaluate liver and kidney function. The kits' instructions, which made sure that the required operating steps were followed, were followed when conducting the experimental procedures. The samples' dilution ratio was changed to satisfy the experiment's particular needs.

**4．H&E Staining of liver, kidney and intestine tissue sections**

The mice were quickly dissected after euthanasia, and the liver, kidneys, and small intestine were quickly separated from the chest and abdominal cavity. The tissues were then carefully cleaned to get rid of blood and fascia. The entire liver and kidneys were then weighed, and the findings were recorded. A portion of the liver, kidney, and small intestine tissues was frozen at -80 °C, while the remainder was preserved by immersion in a 4% (w/v) paraformaldehyde solution. Following the completion of the fixation procedure, the tissues were embedded in paraffin after being dried in ethanol solutions. The tissues were sectioned into 4 μm slices, and then they were stained with H&E for histological examination.

**5. SCFA measurement in mouse feces**

Geng's methodology (1) is rigorously followed in the process for identifying SCFAs in feces. The first step was homogenizing 40 mg of the fecal sample at 40 Hz for 4 min in 1 mL of saturated NaCl solution. The homogenized material was then thoroughly combined with 40 μL of 10% H_2_SO_4_. After that, 800 μL of methyl tert-butyl ether was mixed with 25 mg/L 2-methylvaleric acid to extract SCFAs from the feces using ultrasound for 5 min. For 15 min, the resulting mixture was centrifuged at 10,000 rpm. Following the careful collection of the upper layer of the solution into a centrifuge tube, 0.25 g of anhydrous Na_2_SO_4_ was added to aid in the extraction solution's water absorption. The supernatant was then extracted from the mixture by centrifuging it for 15 minutes at 10,000 rpm. The SCFAs in the supernatant were identified using gas chromatography–mass spectrometry. A 30 m × 250 μm × 0.25 μm HP-FFAP column was purchased from Agilent Technologies Co., Ltd. in Santa Clara, California, USA. The detection was carried out using a Shimadzu QP2020 NX mass spectrometer (Shimadzu (China) Co., Ltd., Shanghai, China). Helium was used as the carrier gas, with an injection volume of 1 μL and a flow rate of 1 mL/min. After being elevated to 80 °C, the temperature was maintained for 1 min. After that, it was heated to 200°C at a rate of 10°C per minute and maintained there for 5 min. At last, it was brought up to 240°C at a rate of 40°C per minute and maintained there for 1 min. The ionization temperature was 200°C.

**Table S1** **Levels of TC, TG, HDL-C, and LDL-C in the serum of Con and Mol group mice**

| Groups | TC/（mmol/L） | TG/（mmol/L） | LDL-C/（mmol/L） | HDL-C/（mmol/L） |
| --- | --- | --- | --- | --- |
| Con | 5.265±0.12 | 2.318±0.25 | 1.500±0.28 | 1.010±0.18 |
| Mol | 7.251±0.28 | 2.744±0.21 | 2.633±0.14 | 0.570±0.12 |

**
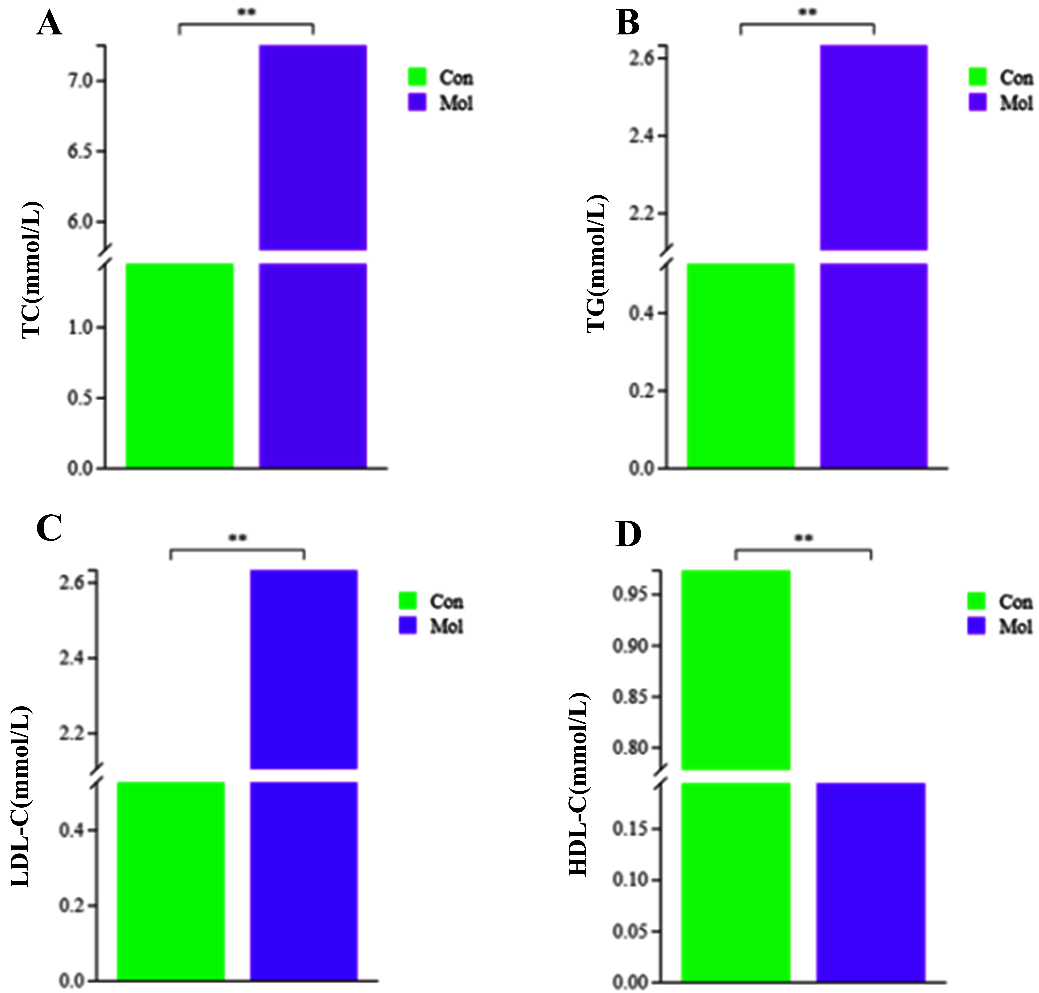
**

Figure S1 Comparison of serum TC(A), TG(B), LDL-C(C), and HDL-C(D) content between of blank and model group mice

**The main ingredients of PSHJ**

Table S2 displays the findings of the analysis of PSHJ's alcohol content, polysaccharides, total acids, and amino nitrogen. Every component satisfies the physical and chemical requirements for dry yellow wine, and the comparatively large polysaccharide content may play a significant role in its physiological activity.

**Table S2 The main ingredients of PSHJ**

| Groups | Total sugar  (g/L) | Polysaccharides  (g/L) | pH | Total Acids  (g/L) | Amino nitrogen (g/L) | Alcohol (Vol %) |
| --- | --- | --- | --- | --- | --- | --- |
| content | 15.510±0.250 | 14.040±0.219 | 4.490±0.105 | 4.167±0.095 | 0.422±0.065 | 21.650±0.088 |

**Table S3 Average daily food intake of each group of mice**

| Groups | Average daily food intake of mice per week（g/mouse） | | | | | | | | | |
| --- | --- | --- | --- | --- | --- | --- | --- | --- | --- | --- |
|  | 1 | 2 | 3 | 4 | 5 | 6 | 7 | 8 | 9 | 10 |
| Con | 9.59 | 7.41 | 6.08 | 6.54 | 6.45 | 6.23 | 5.86 | 5.61 | 5.61 | 5.2 |
| Mol | 10 | 4.72 | 4.54 | 6.07 | 5.74 | 5.59 | 5.32 | 5.78 | 5.24 | 5.39 |
| PTHJ | 10 | 4.51 | 4.87 | 5.98 | 5.31 | 6.02 | 4.98 | 4.24 | 3.52 | 3.87 |
| Low | 9.23 | 3.72 | 3.93 | 5.61 | 4.66 | 4.76 | 3.16 | 3 | 2.79 | 2.84 |
| Mid | 10 | 4.7 | 5.03 | 5.47 | 4.83 | 5.21 | 4.22 | 3.93 | 3.47 | 3.92 |
| High | 10 | 4.46 | 4.35 | 4.67 | 4.17 | 5.42 | 3.77 | 3.57 | 3.6 | 3.42 |

Note: The numbers 1-10 in the header indicate the number of weeks. One week is the adaptive feeding week, 2-6 weeks are the modeling stage, and 7-10 weeks are the administration stage.

**Table S4 Average body weight of each group of mice**

| Groups | Average body weight of mice per week（g/mouse） | | | | | | | | | |
| --- | --- | --- | --- | --- | --- | --- | --- | --- | --- | --- |
|  | 1 | 2 | 3 | 4 | 5 | 6 | 7 | 8 | 9 | 10 |
| Con | 29.42 | 37.85 | 40.58 | 42.13 | 43.51 | 44.98 | 46.78 | 47.22 | 47.59 | 45.68 |
| Mol | 29.03 | 38.63 | 41.27 | 44.15 | 45.74 | 47.75 | 48.17 | 48.5 | 49.1 | 50.2 |
| PTHJ | 29.8 | 39.46 | 43.27 | 45.63 | 46.33 | 49.21 | 50.19 | 50.51 | 49.86 | 49.15 |
| Low | 29.96 | 38.24 | 42.97 | 44.88 | 46.71 | 48.56 | 49.68 | 48.5 | 48.36 | 49.37 |
| Mid | 29.14 | 37.65 | 41.95 | 43.6 | 44.96 | 45.41 | 47.37 | 47.53 | 45.4 | 46.45 |
| High | 30.2 | 40.04 | 43.68 | 45.86 | 47.55 | 49.38 | 51.51 | 51.62 | 49.75 | 48.41 |

Note: The numbers 1-10 in the header indicate the number of weeks. One week is the adaptive feeding week, 2-6 weeks are the modeling stage, and 7-10 weeks are the administration stage

**Table S5 Average FGB values of each group of mice**

| Groups | FBG concentration per week（mmol/L） | | | | |
| --- | --- | --- | --- | --- | --- |
|  | 6 | 7 | 8 | 9 | 10 |
| Con | 5.8 | 5.74 | 5.6 | 5.67 | 5.85 |
| Mol | 8.12 | 8.3 | 8 | 8.3 | 8.48 |
| PTHJ | 8.08 | 9.12 | 7.76 | 7.19 | 7.5 |
| Low | 7.75 | 8.7 | 7.62 | 7.38 | 7.36 |
| Mid | 8.17 | 7.8 | 7.35 | 6.12 | 6.7 |
| High | 8.3 | 8.1 | 7.09 | 6.3 | 6.1 |


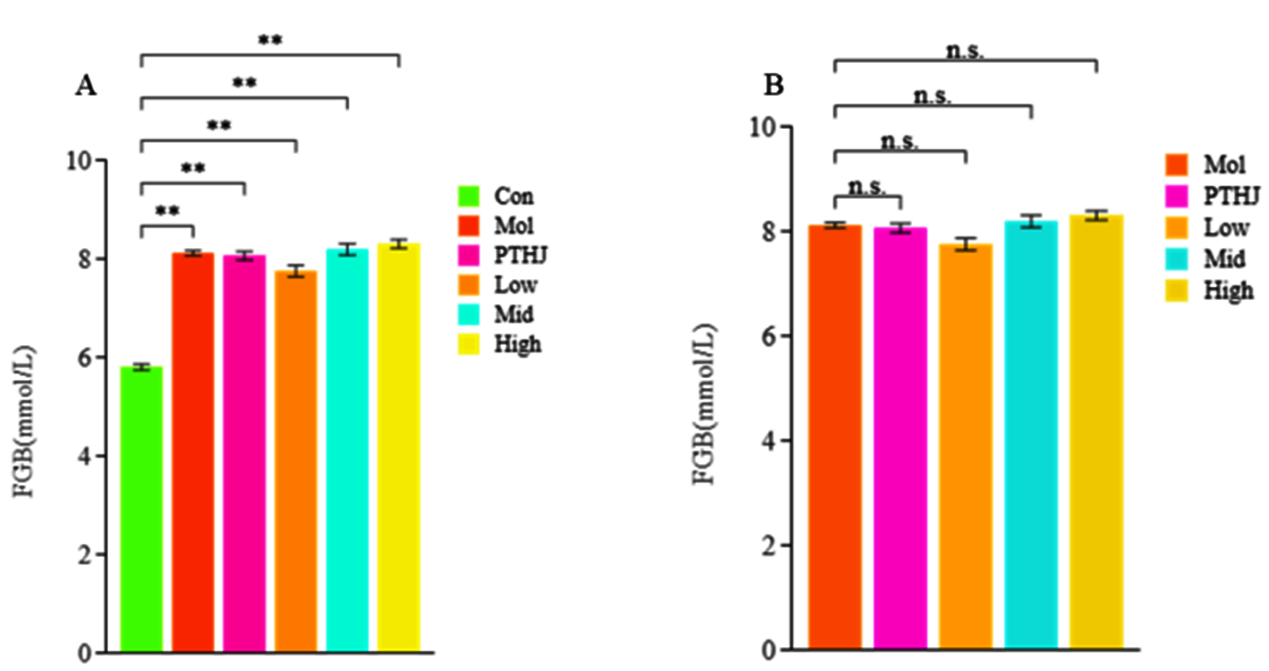


**Figure S2** FGB values of each group of mice at the sixth week.

**Table S6 Serum TC, TG, LDL-C, and HDL-C in each group of mice**

| Groups | Concentration（mmol/L） | | | |
| --- | --- | --- | --- | --- |
|  | TC | TG | LDL-C | HDL-C |
| Con | 1.547±0.01 | 0.670±0.03 | 1.500±0.03 | 1.088±0.05 |
| Mol | 2.544±0.04 | 1.184±0.04 | 4.631±0.02 | 0.389±0.01 |
| PTHJ | 2.212±0.02 | 0.872±0.01 | 3.733±0.03 | 0.637±0.02 |
| Low | 2.116±0.06 | 0.949±0.01 | 3.867±0.02 | 0.652±0.01 |
| Mid | 1.926±0.01 | 0.843±0.01 | 2.730±0.03 | 0.733±0.02 |
| High | 1.842±0.01 | 0.723±0.01 | 2.532±0.03 | 0.853±0.01 |


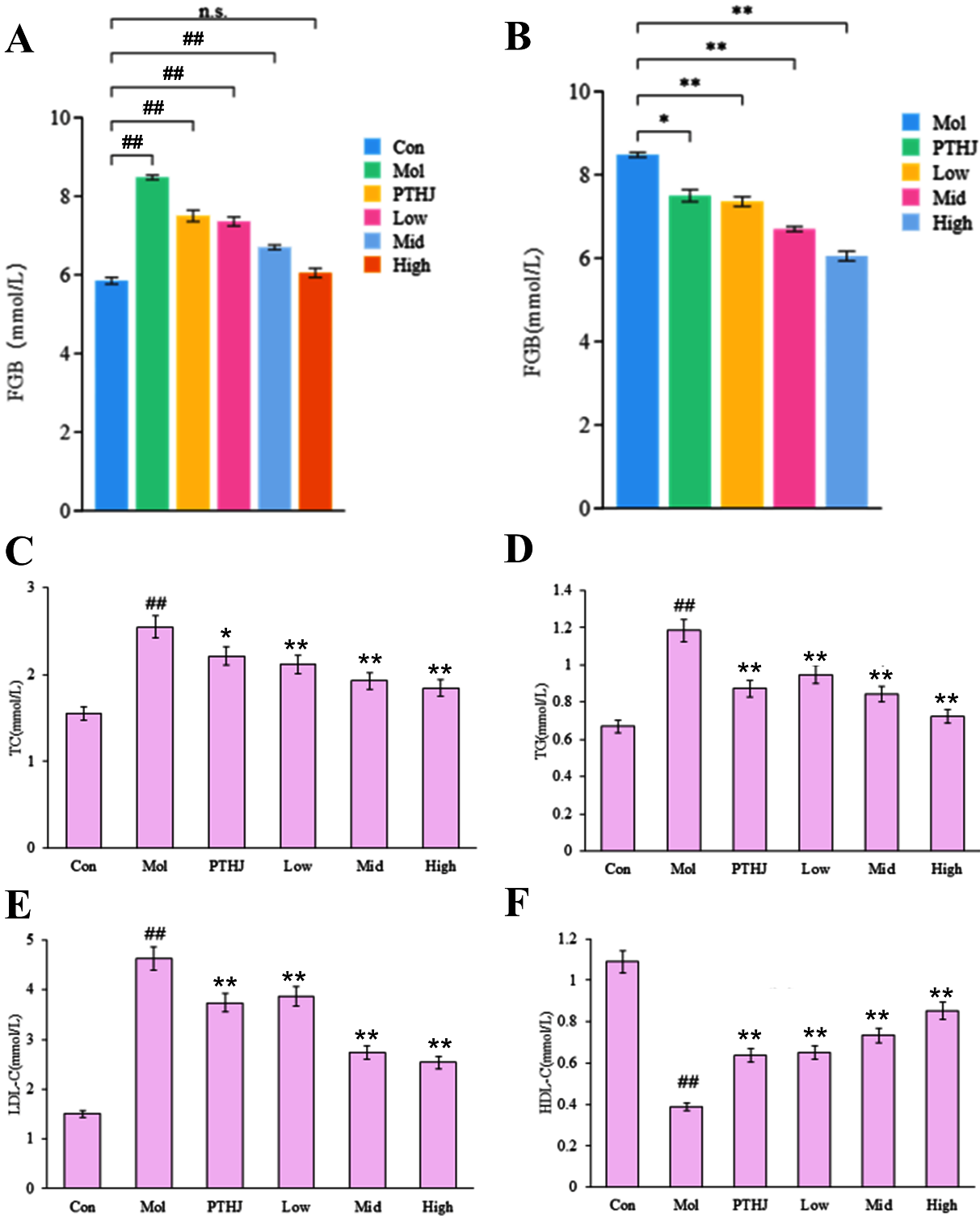


**Figure S3** Effect of PSHJ on glucose and lipid metabolism of mouse. (A) FBG versus the normal control group; (B)FBG versus the model group; (C)TC; (D) TG; (E) LDC-C; (F) HDL-C. Con: normal control group; Mol: model group; PTHJ: ordinary Huangjiu group; Low: low-dose Polygonatum sibiricum Huangjiu group; Mid: medium dose Polygonatum sibiricum Huangjiu group; High: high-dose Polygonatum sibiricum Huangjiu group. ##p<0.01 versus the normal control group. *p<0.05, **p<0.01 versus the model group.

**Table S7 Average organ index of each group of mice**

| Groups | liver index/% | kidney index/% | epididymal fat index/% |
| --- | --- | --- | --- |
| Con | 2.506±0.100 | 0.934±0.011 | 1.303±0.016 |
| Mol | 3.564±0.153 | 1.509±0.024 | 3.891±0.016 |
| PTHJ | 3.099±0.010 | 1.169±0.01 | 3.042±0.058 |
| Low | 3.132±0.032 | 1.128±0.074 | 2.872±0.105 |
| Mid | 2.923±0.022 | 1.077±0.022 | 2.206±0.081 |
| High | 2.751±0.02 | 1.001±0.016 | 1.86±0.08 |


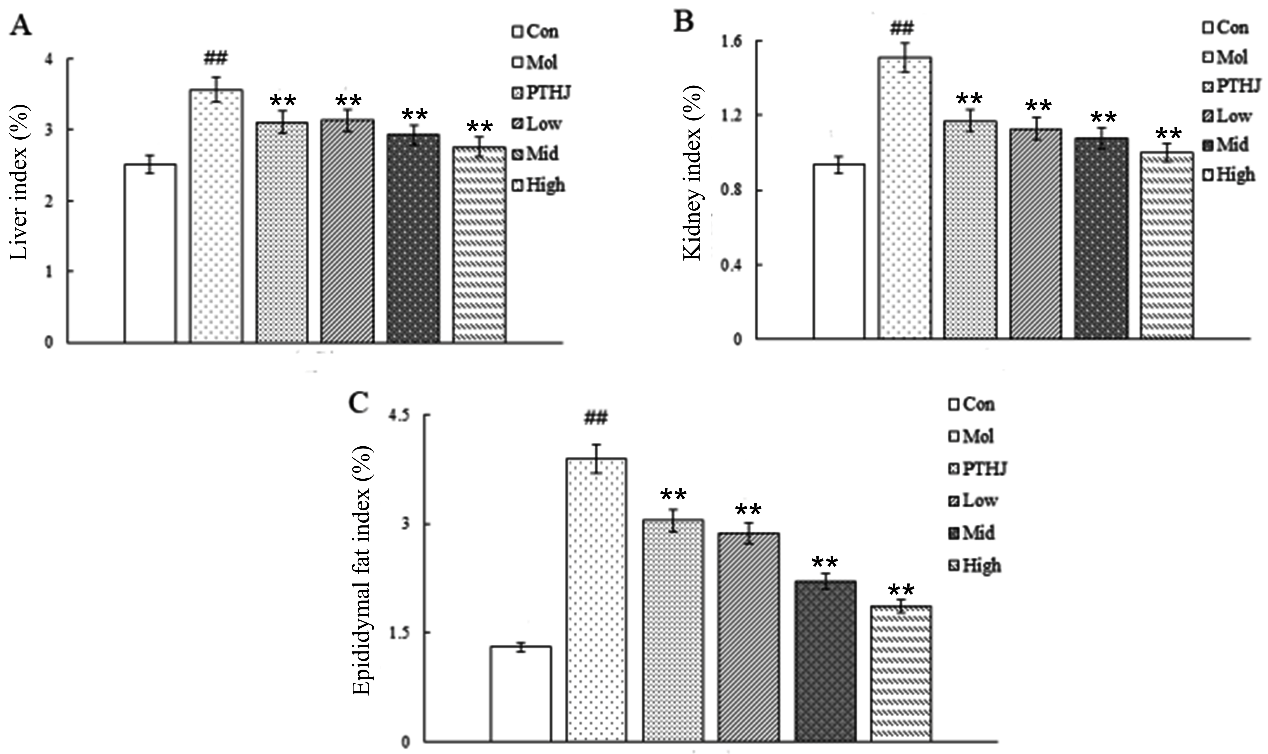


**Figure S4** Comparative analysis of the significance of organ index in each group of mice. ^##^p<0.01 versus the normal control group. *p<0.05, **p<0.01 versus the model group.

| Groups | AST(U/L) | ALT(U/L) | BUN(μmol/L) | CRE(μmol/L) |
| --- | --- | --- | --- | --- |
| Con | 5.009±0..451 | 4.453±0.028 | 0.714±0.011 | 57.370±0.981 |
| Mol | 16.704±0.339 | 19.802±0.383 | 1.571±0.032 | 137.889±1.471 |
| PTHJ | 9.532±0.254 | 8.841±0.156 | 1.189±0.031 | 96.734±2.082 |
| Low | 12.05±0.150 | 10.319±0.159 | 1.083±0.060 | 107.896±1.830 |
| Mid | 9.567±0.249 | 7.451±0.200 | 0.921±0.023 | 83.935±0.770 |
| High | 7.089±0.067 | 5.064±0.101 | 0.82±0.022 | 64.737±0.531 |

**Table S8. Effect of PSHJ on liver and kidney function indicators of hyperlipidemic mice.**

**Table S9 Sample sequencing quantity statistics (bacteria)**

| Groups | Primer | Number of sequences | Base number/unit | Average length/bp |
| --- | --- | --- | --- | --- |
| Con | AGCGCT | 67813 | 25351098 | 373.84 |
| Mol | GCACTT | 61152 | 22816925 | 373.12 |
| PTHJ | GTCATT | 81256 | 30353540 | 373.55 |
| Low | ACGTTA | 77893 | 29056868 | 373.04 |
| Mid | TCTCAC | 60998 | 22735101 | 372.72 |
| High | ACTACC | 72059 | 26893883 | 373.22 |

**Table S10 Sample sequencing quantity statistics (fungi)**

| Groups | Primer | Number of sequences | Base number/unit | Average length/bp |
| --- | --- | --- | --- | --- |
| Con | ACGCTCG | 64429 | 21496353 | 333.64 |
| Mol | AGACGCA | 74749 | 27526900 | 368.26 |
| PTHJ | CGTGTCT | 36952 | 11707797 | 316.84 |
| Low | AGCACTG | 34430 | 11814241 | 343.14 |
| Mid | ATCAGAC | 36425 | 12423494 | 341.07 |
| High | ATATCGC | 74182 | 27919316 | 376.36 |


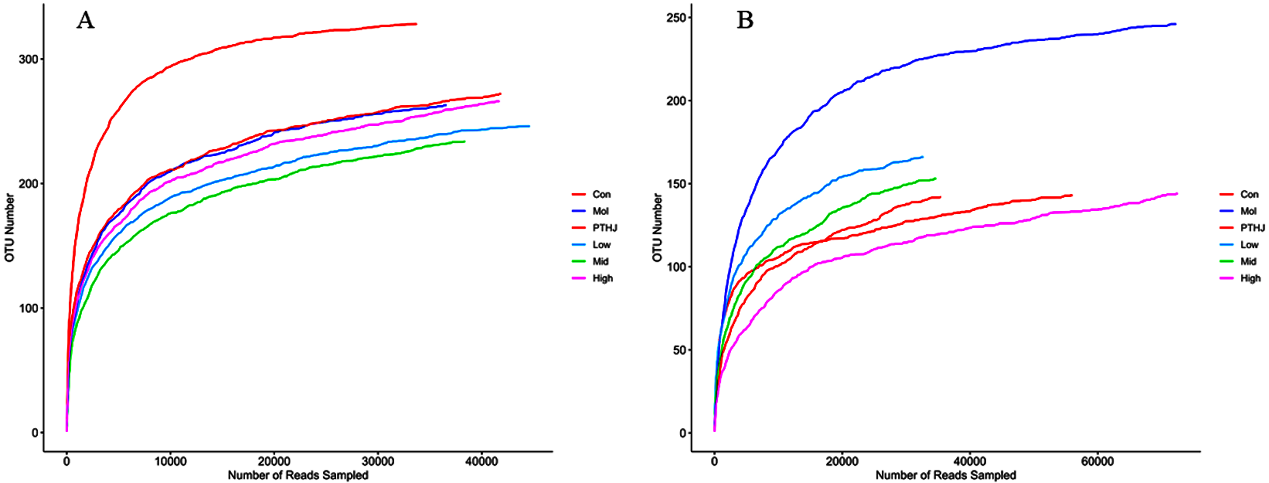


**Figure S5** Alpha diversity dilution curve of gut bacteria (A) and fungi (B) in each group of mice.

**Table S11 Alpha diversity index of different samples (bacteria)**

| Groups | Shannon | Chao | Ace | Simpson |
| --- | --- | --- | --- | --- |
| Con | 4.455 | 336.5 | 334.1 | 0.023 |
| Mol | 3.203 | 256.7 | 259.9 | 0.084 |
| PTHJ | 3.593 | 291.9 | 279.2 | 0.076 |
| Low | 3.519 | 302.3 | 300.9 | 0.076 |
| Mid | 3.732 | 304.8 | 304.6 | 0.043 |
| High | 3.908 | 311.0 | 312.1 | 0.041 |

**Table S12 Alpha diversity index of different samples (fungi)**

| Groups | Shannon | Chao | Ace | Simpson |
| --- | --- | --- | --- | --- |
| Con | 1.855 | 273.2 | 257.0 | 0.331 |
| Mol | 0.862 | 166.2 | 172.8 | 0.731 |
| PTHJ | 1.223 | 171.5 | 180.7 | 0.677 |
| Low | 1.199 | 177.0 | 176.5 | 0.654 |
| Mid | 1.438 | 183.5 | 192.8 | 0.435 |
| High | 1.743 | 192.4 | 216.5 | 0.409 |

**Table S13 Bray distance matrix between different sample groups (bacteria)**

| Groups | Con | Mol | PTHJ | Low | Mid | High |
| --- | --- | --- | --- | --- | --- | --- |
| Con | 0 | 0.344 | 0.291 | 0.285 | 0.253 | 0.239 |
| Mol | 0.344 | 0 | 0.200 | 0.338 | 0.375 | 0.445 |
| PTHJ | 0.291 | 0.200 | 0 | 0.201 | 0.339 | 0.387 |
| Low | 0.285 | 0.338 | 0.201 | 0 | 0.121 | 0.275 |
| Mid | 0.253 | 0.375 | 0.339 | 0.121 | 0 | 0.205 |
| High | 0.239 | 0.445 | 0.387 | 0.275 | 0.205 | 0 |

**Table S14 Bray distance matrix between different sample groups (fungi)**

| Groups | Con | Mol | PTHJ | Low | Mid | High |
| --- | --- | --- | --- | --- | --- | --- |
| Con | 0 | 0.05 | 0.037 | 0.033 | 0.03 | 0.02 |
| Mol | 0.05 | 0 | 0.011 | 0.013 | 0.014 | 0.019 |
| PTHJ | 0.037 | 0.011 | 0 | 0.009 | 0.012 | 0.03 |
| Low | 0.033 | 0.013 | 0.009 | 0 | 0.004 | 0.02 |
| Mid | 0.03 | 0.014 | 0.012 | 0.004 | 0 | 0.02 |
| High | 0.02 | 0.019 | 0.03 | 0.02 | 0.022 | 0 |

**Table S15 Content of SCFAs in feces of different groups of mice**

| Groups | SCFAs (μg/g) | | | | | | | |
| --- | --- | --- | --- | --- | --- | --- | --- | --- |
|  | Acetic acid | Propionic acid | Isobutyric acid | Butyric acid | Valproic acid | Isovaleric acid | Hexanoic acid | Heptanoic acid |
| Con | 386.28±6.7 | 125.37±3.0 | 14.76±0.94 | 79.11±0.83 | 20.55±1.26 | 11.67±0.75 | 0.91±0.04 | 0.039±0.001 |
| Mol | 97.51±5.33 | 17.00±1.66 | 2.89±0.33 | 0.74±0.02 | 2.89±0.29 | 2.14±0.30 | 0.02±0.01 | NA±0 |
| PTHJ | 128.00±3.46 | 25.37±1.01 | 5.70±0.46 | 3.80±0.26 | 3.48±0.10 | 2.90±0.47 | 0.05±0.015 | 0.004±0.003 |
| Low | 122.44±2.12 | 26.77±0.99 | 4.74±0.15 | 11.95±1.02 | 4.85±0.18 | 2.95±0.18 | 0.32±0.06 | 0.015±0.002 |
| Mid | 152.00±3.66 | 29.04±1.04 | 5.93±0.63 | 33.42±0.40 | 12.33±0.51 | 4.40±0.26 | 0.39±0.08 | 0.032±0.003 |
| High | 259.84±5.97 | 94.90±3.01 | 10.59±0.43 | 51.86±0.68 | 14.68±0.93 | 4.58±0.19 | 0.47±0.08 | 0.036±0.002 |


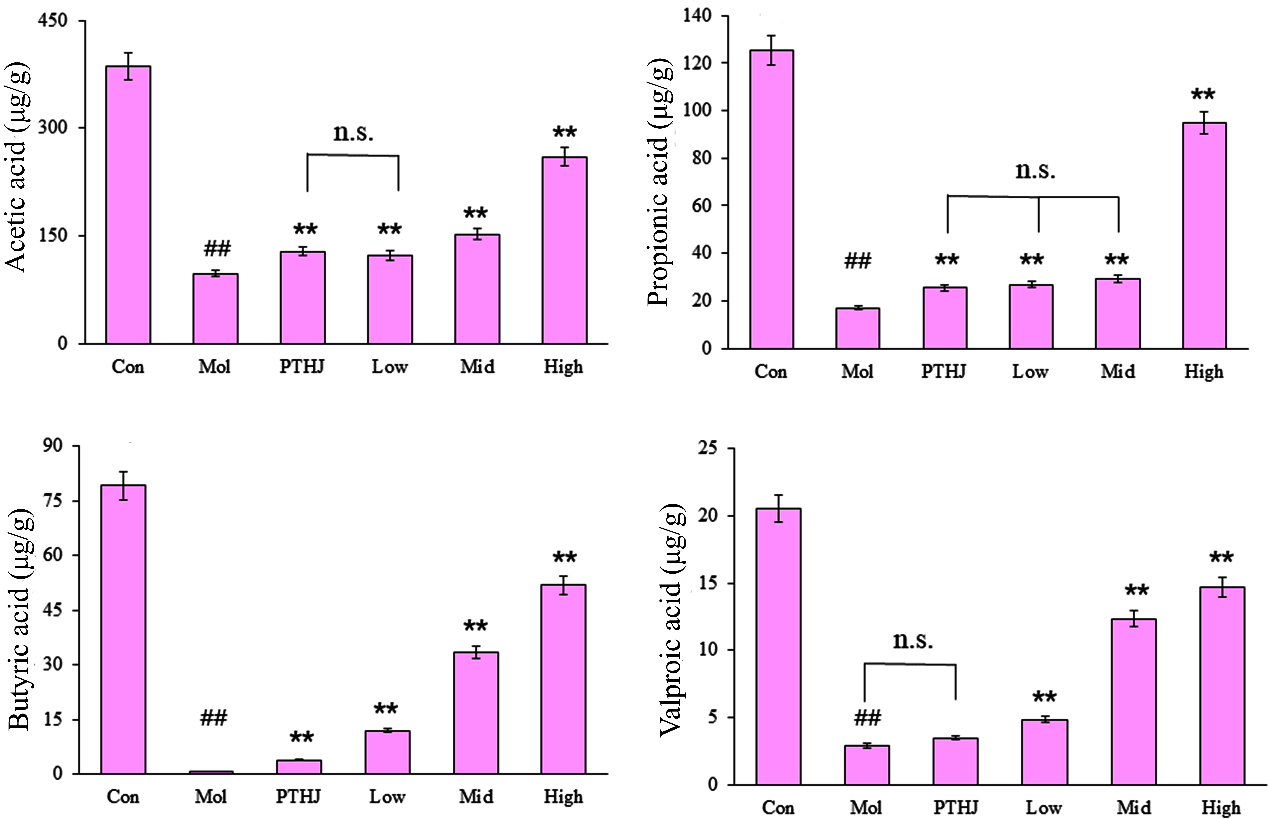


**Figure S6** Comparative analysis of SCFAs content in each group of mice.

^##^p<0.01 versus the normal control group. *p<0.05, **p<0.01 versus the model group.


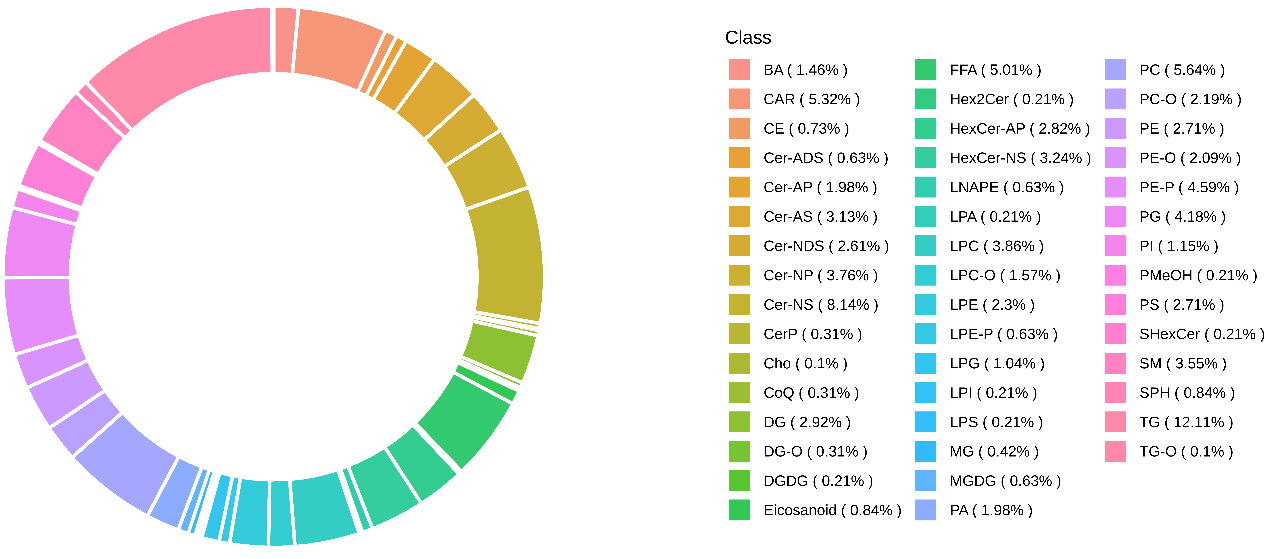


**Figure S7** Lipid subclass ring diagram.


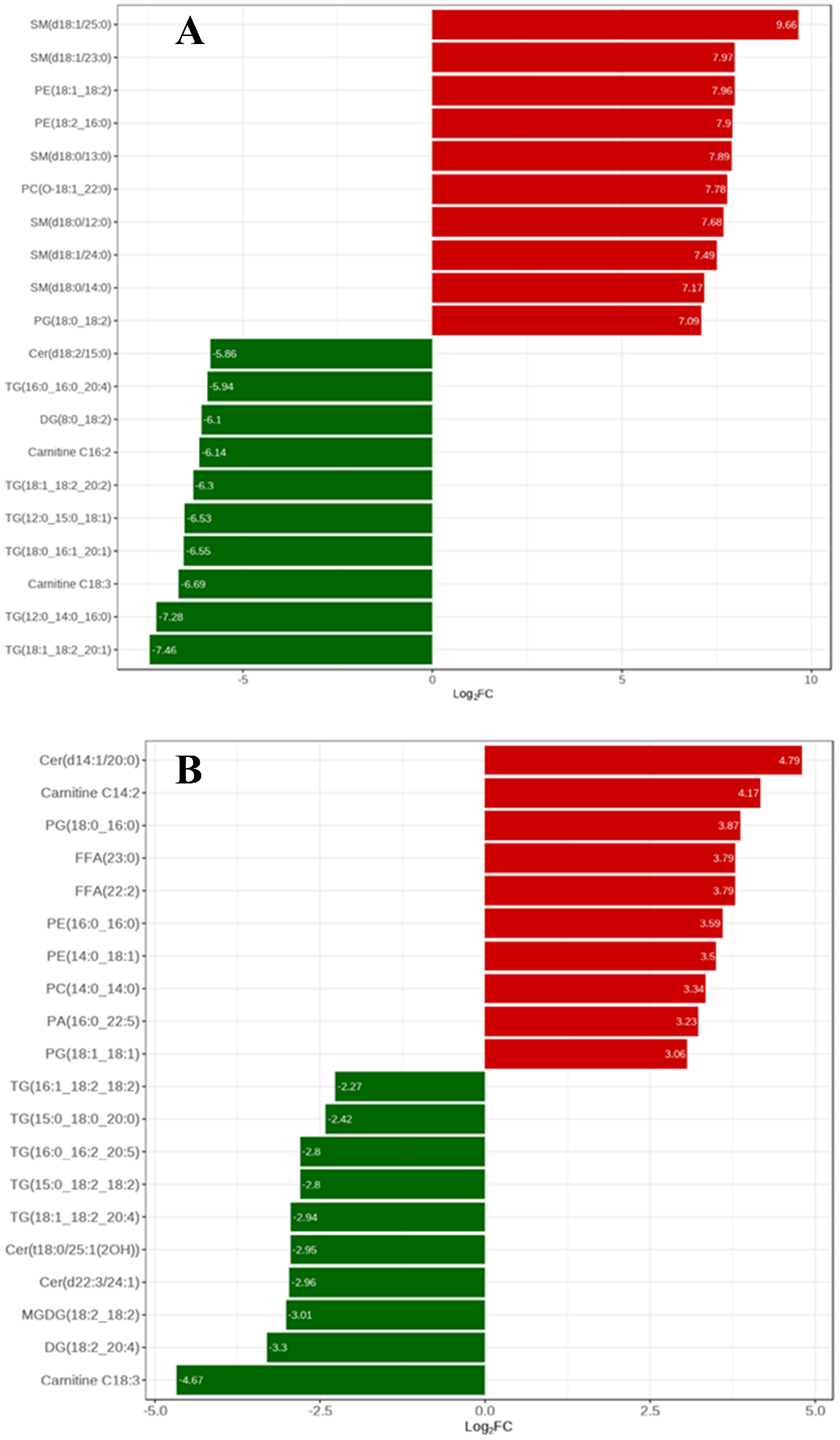


**Figure S8** Ranking of the Top 10 secondary differential lipids with multiple differences between mouse groups. (A) Con vs Mol group; (B) (A) High vs Mol group.


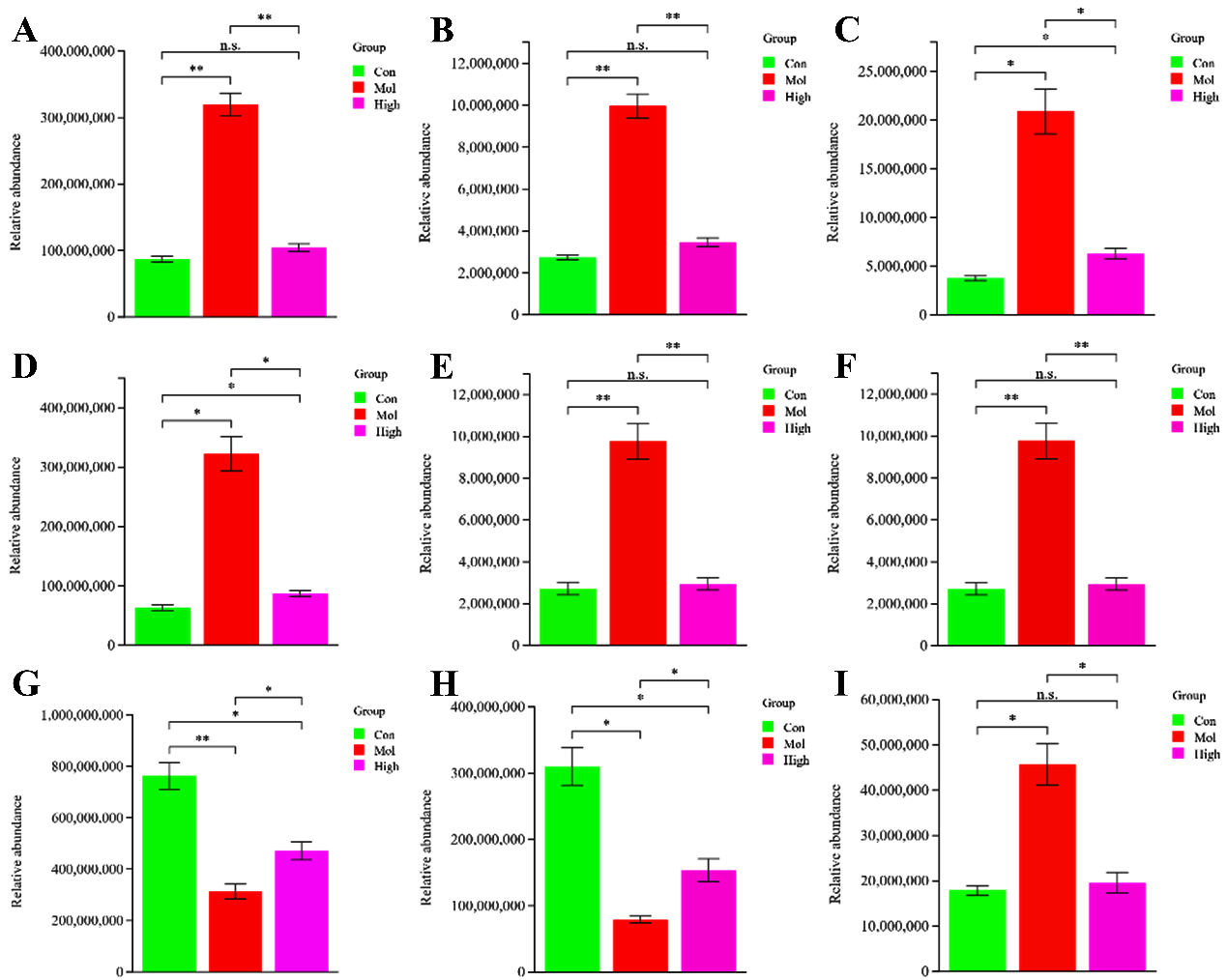


**Figure S9** Comparison of relative abundance of representative differential lipids among different groups of mice. (A) Triglyceride(TG); (B) Diacylglycerol(DG); (C) Phosphatidylethanolamine (PE); (D) Sphingomyelin (SM); (E) Phosphatidylglycerin (PG); (F) Phosphatidylcholine (PC); (G) Ceramide (Cer); (H) Free fatty acids (FFA); (I) Acyl carnitine (CAR). * p<0.05, ** p<0.01, and n.s. indicates no significant differences.

**Reference**

1. Geng J, Wu Y, Tian H, Dong J. Alleviation of high-fat diet-induced hyperlipidemia in mice by *stachys sieboldii miq.* huangjiu via the modulation of gut microbiota composition and metabolic function. *Foods* (2024) 13(15): 2360. doi: 10.3390/foods13152360.
